# Adsorption of Phenoxyacetic Herbicides from Water on Carbonaceous and Non-Carbonaceous Adsorbents

**DOI:** 10.3390/molecules28145404

**Published:** 2023-07-14

**Authors:** Magdalena Blachnio, Krzysztof Kusmierek, Andrzej Swiatkowski, Anna Derylo-Marczewska

**Affiliations:** 1 Faculty of Chemistry, Maria Curie-Sklodowska University, M. Curie-Sklodowska Sq. 3, 20-031 Lublin, Poland; magdalena.blachnio@mail.umcs.pl; 2 Institute of Chemistry, Military University of Technology, Gen. S. Kaliskiego St. 2, 00-908 Warszawa, Poland; krzysztof.kusmierek@wat.edu.pl (K.K.); a.swiatkowski@wp.pl (A.S.)

**Keywords:** carbonaceous adsorbents, inorganic adsorbents, low-cost adsorbents, phenoxyacetic herbicides, herbicides removing, herbicides adsorption

## Abstract

The increasing consumption of phenoxyacetic acid-derived herbicides is becoming a major public health and environmental concern, posing a serious challenge to existing conventional water treatment systems. Among the various physicochemical and biological purification processes, adsorption is considered one of the most efficient and popular techniques due to its high removal efficiency, ease of operation, and cost effectiveness. This review article provides extensive literature information on the adsorption of phenoxyacetic herbicides by various adsorbents. The purpose of this article is to organize the scattered information on the currently used adsorbents for herbicide removal from the water, such as activated carbons, carbon and silica adsorbents, metal oxides, and numerous natural and industrial waste materials known as low-cost adsorbents. The adsorption capacity of these adsorbents was compared for the two most popular phenoxyacetic herbicides, 2,4-dichlorophenoxyacetic acid (2,4-D) and 2-methyl-4-chlorophenoxyacetic acid (MCPA). The application of various kinetic models and adsorption isotherms in describing the removal of these herbicides by the adsorbents was also presented and discussed. At the beginning of this review paper, the most important information on phenoxyacetic herbicides has been collected, including their classification, physicochemical properties, and occurrence in the environment.

## 1. Introduction

The production of pesticides is an important branch of the chemical industry, and their use for crop protection and pest elimination is a common and necessary practice used throughout the world. The consumption of crop protection products has increased many times over the years. In 2020, their annual consumption in agriculture was estimated at 2.7 million tons, of which about 52% are herbicides, 23% are fungicides, 18% are insecticides, and 7% are other pesticides [1]. Today, the most commonly used herbicide is glyphosate, which is the main ingredient in the popular market preparation called Roundup. Apart from glyphosate, the most popular and widely used herbicides are those derived from phenoxyacetic acid, especially 2,4-dichlorophenoxyacetic acid (2,4-D) and 2-methyl-4-chlorophenoxyacetic acid (MCPA) are used on a mass scale. In 2014, the US Environmental Protection Agency (EPA) approved the combined use of glyphosate and 2,4-D, a mixture available under the trademark “Enlist Duo”. In January 2022, the EPA [2] renewed the limited-time registration of this product, which is expected to contribute to the even greater use of both herbicides. Phenoxyacetic herbicides act as synthetic auxins and growth regulators and are widely used to control broadleaf weeds in farmlands, pastures, lawns, and grassy rights of way. The widespread use of these herbicides is associated with their massive release into the surface and groundwater. Their presence in the aquatic environment is undesirable, especially since these compounds are characterized by quite high toxicity to living organisms [3,4,5]. Therefore, their degradation and removal from the aquatic environment and prevention of their entry into the ecosystem become a priority issue.

In recent years, many methods have been proposed for the removal of chlorophenoxy herbicides from water. In general, these methods can be divided into three main groups: biological methods, chemical methods, and physical methods [6,7,8,9,10]. The most conventional treatment techniques are as follows: biodegradation, irradiation, oxidation, ozonation, chemical coagulation-flocculation, electrokinetic coagulation, thermal remediation, ion exchange, membrane filtration, and adsorption. Although some of them seem to be effective for chlorophenoxy herbicides removal, closer insight reveals several disadvantages such as: the production of biosolids that may cause eutrophication of water bodies, the use of chemical catalysis, the use of chemicals, requirement of oxygen or ozone atmosphere, requirement of the pre-treatment stage, high energy consumption, risk of membrane fouling, generation of sludge or harmful by-products, and high cost. Adsorption deserves special attention, which turns out high effective, low cost, and low energy-intensive technique for wastewater treatment; moreover, the purification process does not require chemicals, does not produce additional pollutants, and is easy to implement.

In the present review, an overview of different materials used as conventional and non-conventional adsorbents for phenoxy herbicide removal from water is presented. In addition to the traditionally used activated carbons, a large pool of adsorbents consists of inorganic materials mainly those based on silica, various types of unconventional alternative adsorbents such as natural and waste materials, and more advanced materials such as inorganic-organic composites or doped carbons. These latter solids show good adsorption efficiency towards herbicides and other organic compounds as well [11,12,13,14].

The review presents a critical analysis of these materials; literature has been discussed in terms of adsorption capacities, fitted isotherm and kinetic models, and other aspects. We hope that such a review will help select the right adsorbent in designing and synthesizing new materials with better adsorption properties.

## 2. Physical, Chemical, and Biological Properties of Phenoxy Carboxylic Acid Herbicides

Chlorophenoxy herbicides belong to the class of aryloxyalkanoic acids that are derivatives of 1–3 carbon hydroxyalkanoic acids with aromatic substituent attached to the alcoholic oxygen. They were developed in the 1940s to synthesize analogues of the auxin, indole-3 acetic acid (IAA), playing the role of a natural plant growth regulator. The mechanism of action of this group of chemicals (also called as auxinic herbicides) is based on mimicking IAA at the molecular level, which controls cell enlargement, division, and plant growth through the plant life cycle [15].

Generally, it is assumed that chlorophenoxy herbicide interacts with auxin-binding proteins (ABPs) located in the cell membrane, endoplasmic reticulum, and cell nucleus, and causes similar physiological effects to a natural IAA [16]. However, as far as IAA concentration is regulated by synthesis, degradation, and conjugation to other molecule processes in a plant, in the case of auxin-like herbicide, its amount is too large to be controlled by the plant regulation system. Herbicide mobilizes metabolic reserves that are transported to the site of growth in meristematic tissue. Consequently, it comes down to twisting and elongation of leaves and stems, damaging repair mechanisms, and, finally, plant death.

In terms of biochemistry, a plant treated with herbicide shows inhibition in respiration and photosynthesis processes due to the degradation of chlorophyll. The first symptoms of the herbicidal activity appear within a few hours from the moment of application. The effect of lethally abnormal growth is far less marked in grasses than in other species, hence chlorophenoxy acids are recognized as selective herbicides. Generally, the herbicidal activity of this group reveals towards dicotyledonous plant species while monocotyledonous ones remain relatively unaffected [17,18]. For this reason, auxinic herbicides are used to control broad-leaved annual and perennial weeds in agricultural and non-agricultural areas. Chlorophenoxy herbicides are usually applied post-emergence, alone, or combined with other herbicides. The last reports show [19,20,21] that chlorophenoxy group representatives—2,4-D and MCPA are at the top of the most commonly used pesticide-active ingredients in the US and the EU.

An active form of chlorophenoxy herbicides is acid but, commercially, these herbicides are formulated as esters, amines, choline salts, and salts with alkali metals. The type of formulation affects the method of application, the physicochemical properties of herbicide in the environment, and its ability to penetrate the plant. Because chlorophenoxy esters are soluble in organic solvents and oils while insoluble in water, they are used as aqueous emulsions with a suitable emulsifying agent. A great advantage of the ester formulation is resistance to washing from the leaves following rain. Nowadays, the production of methyl, ethyl, and isopropyl esters is limited because of their high vapor pressure and ability to volatilize, leading to the destruction of the surrounding crops. Ultimately, low molecular esters were replaced by ones with larger side chains that reduce their volatility [22]. The high solubility of esters in the plants’ cuticle affects more efficient absorption of them by plants, and after conversion to the corresponding acid, greater herbicidal activity is observed. For this reason, the ester formulation is recognized as a better weed controller than others. The herbicide’s residues that were not absorbed by the plant readily hydrolyze to their acid forms in the environmental conditions.

The amines and salts are readily soluble in water, which makes them vulnerable to being washed from leaves by rain. They are also characterized by low efficiency at moving from the leaf surface across the cuticle and into the plant. These forms of herbicides dissociate in water and form anions of the corresponding acids.

Because of the hydrolytic or dissociative abilities of the different formulations, the acid forms of chlorophenoxy herbicides are most often described in the studies on the mode of their action in living organisms. There are also many investigations on the chemical and physical processes they undergo in environmental systems. The pKa values of chlorophenoxyacetic acids are in the range of 2.56–3.36, so as weak acids, they dissociate into ions in water within the normal pH range of soils and environmental waters. They are slightly soluble in water and readily soluble in organic solvents, as indicated by the values of K_ow_, which depend on the chemical properties of the compound, i.e., quantity, type of functional groups, their position on an aromatic ring, and the length of hydrocarbon residue in a molecule. The solubility of weak acids depends on the solution’s pH. Both solubility and the weak-acid nature of herbicides are the main factors that influence their uptake by plants and translocation within their tissues.

The physical and chemical properties of phenoxyacetic acid herbicides (4-chlorophenoxy acetic acid, 4-CPA; 2,4-dichlorophenoxy acetic acid, 2,4-D; 2,4,5-trichlorophenoxy acetic acid, 2,4,5-T; 4-chloro-2-methylphenoxy acetic acid, MCPA) are presented in Table 1.

## 3. Chlorinated Phenoxyacetic Acids in the Natural Environment

The presence of chlorinated phenoxyacetic acids residues in the natural environment is the consequence of their direct application in agricultural and urban areas. The problem concerns diverse constituents of the environment: atmosphere, soil, and water.

Volatilization during application plays the main role in getting the chemical compounds into the air. Another process taking place is the evaporation of herbicide from soil and plant surfaces. The volatility of herbicide is strictly determined by the type of formulation. Generally, the ester formulations have a greater ability to volatilize than the salt ones. For this reason, the production of esters with high volatility was prohibited and replaced by products that were more environmentally friendly. This is the molecular weight that determines the volatile ability of the esters.

There are some other factors and additives that affect the herbicide volatility, such as temperature, soil moisture level, organic matter and clay content in the soil, and adjuvant in the formulation. The increase in the first two factors intensifies volatility while the others weaken the process [22,32,33].

Being in the air, the phenoxy herbicide can be subjected to photolysis, photodegradation with hydroxyl, ozone, and nitrate radicals, or aqueous oxidation in aerosols [34]. Furthermore, some molecules can fall on the surrounding surface as a dry deposit or after dissolving in water droplets as a wet deposit [35,36]. The atmospheric lifetime of a given herbicide depends on its structure and the rate of the chemical and physical processes it undergoes. Depending on the weather conditions and herbicide form (vapour, droplets, and suspension with soil and dust), it may be transported in the air for a certain distance from the application location [37].

The studies conducted in 2019 in Germany indicated the MCPA detection frequency in the atmosphere at a level of 44.9% and a concentration range from 10 to 90 ng/sample [38]. In 2020, similar results with the minimal, maximal, and mean concentrations were obtained: 12.0, 71.0, and 30.9 ng/sample, respectively, were noted for 2,4-D in Austrian cities and agricultural areas. Here, the presence of the herbicide in 93% of the samples was confirmed [39]. In turn, according to the report of the air monitoring program in France (the Provence-Alpes-Côte-d’Azur region and Corsica) conducted over the years 2012–2017, the detection frequency of 2,4-D and MCPA was only 2% from 726 samples collected from rural and urban areas. The maximal concentrations for 2,4-D and MCPA were 2.69 and 0.54 ng m^−3^, respectively [40].

The presence of chlorinated phenoxyacetic acids in the soil as a non-target element of the environment is related to their translocation from the atmosphere or plant surface treated with herbicide. Being in soil, a herbicide may undergo some possible processes, such as adsorption, migration to the aqueous phase, or degradation.

The herbicide adsorption by soil enables the retention of a chemical compound in a solid phase, which plays a role of a natural filter and reservoir. At the same time, adsorption decreases both herbicide migration into the soil solution as well as the rate of volatilization, bioavailability, and biodegradation, because the latter processes usually take place from the aqueous phase. In general, adsorption is reversible while the soil capacity depends on many factors, such as herbicide properties, pH, texture, moisture of soil, organic matter and clay content, temperature, and intensity of rainfall.

Chlorinated phenoxyacetic acids are weak in nature, occurring in the form of anionic species within the normal pH range of most soils (pH 5–8), but as pH decreases, they undergo the protonation reaction. Typically for acidic herbicides, more molecular species are formed more effectively adsorbed by soil [41,42]. An increase in soil acidity can result in the significant contribution of organic matter (peatbogs) or a rapid downward flow of rainwater that can leach cations out from the soil surface layers following ion exchange. On the other hand, if the soil pH is relatively high, the herbicide’s anionic form is predominant, and, thus, repulsion between the negatively charged molecules and the negatively charged fraction of organic matter occurs. The adsorption based on the electrostatic forces is also related to clay-rich soils [43,44,45]. However, adsorption increases only when the ambient pH is below the point of zero charge (pH_pzc_) of the clay soil, and attraction between herbicide-deprotonated molecules and positively charged surface sites on minerals occurs. Due to high pH_pzc_ values and, what is important, large surface areas of iron oxyhydroxides, they work well as adsorbents for chlorophenoxy acids [46].

Based on the parameter of distribution coefficient between soil and aqueous solution (K_d_) and the partitioning coefficient between soil organic carbon and aqueous solution (K_oc_), many studies have showed that chlorinated phenoxyacetic acids are weakly adsorbed in most of the agricultural soils and sediments [47,48,49,50,51]. Such weak interaction enhances herbicide mobility in soil, carrying a threat of leaching it out into surface water or even groundwater. However, its mobility to water systems is also affected by dynamics of degradation comprising chemical, photochemical, or biological processes in the upper soil horizons [52]. 

Due to the high agricultural and non-agricultural usage of chlorinated phenoxyacetic acids in the world, they are real pollutants of surface waters. The problem concerns all of Europe, where the public water supply of 37% is sourced from surface waters (rivers, lakes, and impounding reservoirs) [53]. In some countries, such as Bulgaria, Ireland, the UK, and Greece, the contribution of surface water resources for domestic purposes is much higher, and is over 65% [54,55,56,57,58]. In Northern Ireland, surface water is almost the only source of drinking water [59].

The problem, albeit to a lesser extent, affects the countries where groundwater resources are commonly used. The presence of herbicides in groundwater can result from their migration through runoff and infiltration or spillage. Additionally, their weak adsorption capability to most soils and high mobility in the aquatic biotope contribute to the risk of groundwater pollution. The concentration of the chlorinated phenoxyacetic acids in groundwater is dependent on the frequency of agrotechnical practices, soil, climatic, and geological factors. The European Union (EU) established a maximum concentration of 0.1 μg L^−1^ and 0.5 μg L^−1^ for the single pesticide (or its metabolite) and the sum of all pesticides in drinking water, respectively [60]. In turn, the U.S. EPA set the maximum concentration of a single pesticide in drinking water at 70 μg L^−1^.

The scale of the problem may be evidenced by the results of monitoring the state of water contamination with organic compounds. MCPA was found in over 8% of 845 samples from Irish groundwater [61] and in 7% of samples from 112 Spanish groundwater wells [62]. 4-chloro-2-methylphenol—a metabolite of MCPA—was in 28% of the Irish groundwater samples [63]. Due to the high solubility and leachability of salts of MCPA formulations, the worse data refer to surface waters. The herbicide was detected in 93% of 68 samples collected in different sites along the Danube river [64] and 33% of samples from five streams in Switzerland [65].

In Ireland, the 2,4-D concentration of the groundwater samples collected from seven locations ranged from 0.002 to 0.007 μg L^−1^, with a mean value of 0.001 μg L^−1^ [66], while 2,4-dichlorophenol—a metabolite of 2,4-D was detected in 16% of the groundwater samples [63]. In Spain, 2,4-D was noted in 33% of the samples, with concentration ranges of 0.020–0.068 µg L^−1^ and 0.026–0.177 µg L^−1^ for surface water and groundwater, respectively [67]. Data from 19 sites from 16 watersheds across Canada showed that 2,4-D was found in 85% of the 150 prairie and urban river samples. The mean, median, and ranges of concentrations for all samples of herbicide were 172.1 ng L^−1^, 52.7 ng L^−1^, and 0.47–1960 ng L^−1^, respectively. Samples collected after rainfalls had 3-fold higher concentrations of 2,4-D in comparison to ones from the dry season, and over 1.5-fold higher herbicide concentrations were noted in samples from downstream sites in comparison to upstream ones [68].

In natural surface and groundwaters, the degradation rate of chlorinated phenoxyacetic acids depends on the characteristics of the system. For biodegradation, the primary factors concern the content of nutrients and acclimated microorganism populations, temperature, and availability of oxygen. For photolysis, the availability of ultraviolet radiation from sunlight and water clarity plays major roles. The less suspended organic matter in water, the higher the photodecomposition rate of herbicide. Therefore, the herbicide half-life increases with depth due to a reduction in penetration. Considering volatile ester formulations, their dissipation from the aquatic environment can proceed through volatilization or hydrolysis processes. The rate of the vaporization of esters is faster than the hydrolysis rate in neutral and acidic waters [36].

## 4. Adsorption of Chlorinated Phenoxyacetic Acids on Carbonaceous Adsorbents

Activated carbons are common and most frequently used among carbon adsorbents to remove chlorophenoxy herbicides from water. Most of the scientific publications in this field are devoted to them [23,69,70,71,72,73,74,75]. They are mainly commercial granulated or powdered activated carbons. However, research is also carried out with the use of other carbon materials. They include both commercial and laboratory-produced materials. Examples of the former are carbon black and carbon nanotubes, and the latter is graphene or reduced graphene oxide. Compared to activated carbon, the abovementioned carbon materials generally have smaller specific surfaces, a different internal and porous structure, a lower ash content, and a different shape of the external surface. In addition, some of them can be classified as nanomaterials and others are not.

### 4.1. Commercial Activated Carbons

Commercial activated carbons have often been used to remove chlorophenoxy herbicides from water by adsorption. The reason for this is their high adsorption capacity resulting from their highly developed porous structure. These adsorbents are readily available and produced in large quantities. In research, they are used either in the form provided by the manufacturer [23,73,76,77,78,79,80,81,82,83,84,85,86,87] or washed with distilled water at different temperatures or process times [23,70,72,88,89,90,91,92,93]. The reason for doing so is to wash out the water-soluble components of the ash contained in commercial activated carbons. Sometimes, ground ash removal with acids was used [69,71,83,94,95,96,97]. In the case of granular activated carbons, it is also desirable to wash out the pulverized carbon. In some works, modifications of the surface chemistry of commercial activated carbons are used, leading to the formation of oxygen or nitrogen surface functional groups [71,92]. For granulated activated carbons, modifications of the porous structure are also used. This is caused by the abrasion of subsequent layers of granules [73,83]. High-temperature annealing in an oxygen-free atmosphere is also used [96,97]. The influence of the effects of these modifications on the adsorption properties of activated carbons against herbicides in aqueous solutions was analyzed. The idea was to explain the mechanism of the adsorption process. The surface properties of the activated carbons used were precisely characterized. The most commonly used herbicides in the studies were 2,4-D [23,69,70,72,77,79,80,81,82,83,84,85,90,91,92,93,94,95,96,97] and MCPA [69,70,71,72,76,78,79,80,82,88,93] and, somewhat less often, MCPP, MCPB, or others [23,71,73,79,80,81,82,84,88,89,91,92,95,96]. Usually, the adsorption equilibria have been studied [23,69,70,72,73,76,77,78,79,80,81,82,84,85,88,89,90,91,92,93,94,95,96,97], and often the kinetics of the process as well [23,69,70,71,72,73,76,77,78,79,81,83,90,91,92,94,95,96,97]. The most frequently used in research were aqueous solutions of individual herbicides for one pH value and one (room) temperature [23,69,70,73,80,83,85,88,96,97]. Some works used different pH values, different process temperatures, and the addition of other substances, e.g., salt (NaCl, Na_2_SO_4_). The Langmuir and Freundlich adsorption isotherms equations were most often used for the mathematical description of adsorption equilibria. Some of the works also used Sips, Dubinin-Radushkevich, Redlich-Peterson, and Langmuir-Freundlich models. When describing the kinetics of the adsorption process, pseudo-first- (PFO) and pseudo-second-order (PSO) models were most often used. Sometimes the Weber–Morris, Boyd, or multi-exponential models were also used. The results of chlorophenoxy herbicides adsorption studies on commercial activated carbons are listed in Table 2. Analysing the adsorption parameters for commercial activated carbons, certain regularities can be observed. When they are not subjected to any modification, their surface area is in the range of 700–1200 m^2^ g^−1^. The maximum amount of adsorption of 2,4-D or MCPA is generally proportional to their surface area.

### 4.2. Activated Carbons from Solid Wastes

Activated carbons produced from various waste materials have found wide applications in water treatment, including the removal of phenoxyacetic herbicides. These “low-cost activated carbons” are much cheaper than commercially available ACs, and their adsorption abilities are comparable and sometimes even better than commercial adsorbents. Such activated carbons can be divided into two main groups, depending on the origin of the precursor: activated carbons from agricultural waste [98,99,100,101,102,103,104] and activated carbons from synthetic waste [105,106,107,108]. Table 3 summarizes the data of published works.

A series of works published by Hameed et al. [98,99,100,101,102,103,104] describes the preparation of various activated carbons from biological waste and [99] their use as adsorbents to remove pesticides, including 2,4-D, from water. Activated carbons were produced via chemical activation with KOH [98,99,100,102] and H_3_PO_4_ [101,103] or by steam activation [104]. As precursors, date stones [99], oil palm frond [98], corncob [101], banana stalk [100], pumpkin seed hull [102], coconut shell [104], and langsat empty fruit bunch [103] were used. In most of these works, the effects of contact time, initial concentration of 2,4-D, temperature, and pH on the adsorption were all studied. The adsorption kinetic data were analyzed using the PFO and PSO models, while the adsorption isotherms of 2,4-D on the ACs were analyzed using the Langmuir and Freundlich isotherm equations. In general, the adsorption capacities of the activated carbons prepared in these works were comparative with other ACs including the commercially available ACs.

AC from waste slurry was prepared by treating it with hydrogen peroxide and then heated to 200 °C in the air [105]. The activation was performed by heating the sample for 1 h in a muffle furnace at 450 °C in the presence of air. The BET surface area of the product was found to be 710 m^2^ g^−1^. So prepared material was tested for removal of 2,4-D and carbofuran from an aqueous solution. The adsorption of 2,4-D was a second-order process and was controlled by pore diffusion. Carbonaceous adsorbent prepared from carbon slurry exhibited an uptake capacity of 212 and 208 mg g^−1^ for 2,4-D and carbofuran, respectively.

Cansado et al. [107] investigated the removal of MCPA by activated carbons prepared from recycled polyethylene terephthalate (PET) by chemical activation with potassium hydroxide. The material prepared (PET-2-700) has a specific surface area of 1334 m^2^/g, and it was then modified by oxidation with nitric acid (PET-2-700HN), and by reduction at high temperature (PET-2-700T), reduction with NaOH (PET-2-700N), and reduction with urea (PET-2-700U). The ACs exhibited the following order towards the adsorption of MCPA: PET-2-700HN < PET-2-700T < PET-2-700 < PET-2-700N < PET-2-700U.

In another study by Cansado et al. [108], ACs were produced from the particleboard and medium-density fibreboard monoliths by physical activation with CO_2_. These materials were characterized and used as adsorbents for the removal of herbicides, including 2,4-D, MCPA, and diuron. For all ACs, the adsorption efficiency was found to be: diuron < 2,4-D < MCPA.

The same research group [106] evaluated the removal efficiency of 2,4-D and MCPA from water by ACs prepared from synthetic polymers mixtures, such as polyethylene terephthalate (PET) and polyacrylonitrile (PAN), by chemical activation with KOH or K_2_CO_3_ at 800 °C. The obtained ACs exhibited a high specific surface area (from 1206 to 2828 m^2^ g^−1^) and micropore volume (between 0.35 and 1.38 cm^3^ g^−1^). The better results (higher BET area and micropore volume as well as the adsorption capacity towards the herbicides) were observed for the ACs activated with KOH than with K_2_CO_3_. The results revealed that these ACs were much better efficient adsorbents than the commercial activated carbons. The results of chlorophenoxy herbicide adsorption studies on activated carbons produced from solid wastes are summarized in Table 3.

### 4.3. Other Carbonaceous Materials

In one of the papers [109], the authors investigated the adsorption of 2,4-D and MCPA herbicides on six different commercial carbon blacks with A highly differentiated BET-specific surface areas (24.3–137 m^2^ g^−1^) and oxygen content (0.5–2 at.%). They also used activated carbon and graphite for comparative purposes. In [83,110], the adsorption of 2,4-D on commercial carbon black as well as commercial graphitized carbon black with surfaces of 230 and 98 m^2^ g^−1^, respectively, was also investigated. In the first of the above works [110], the carbon blacks were comparative materials with the products of the reaction of hexachlorobenzene and hexachloroethane with sodium azide obtained by the combustion method. Both carbon blacks were chosen because of their similar specific surfaces obtained from C_6_Cl_6_ or C_2_Cl_6_, respectively. In the second work [83], in addition to the two carbon blacks, two carbon molecular sieves and two activated carbons were used for 2,4-D adsorption studies.

In another work [111], the authors investigated the use of unmodified carbon black in their production as well as this carbon black modified (with tannic or gallic acid) in the process of adsorption of the herbicides 2,4-D and MCPA. The unmodified carbon black turned out to be a better adsorbent for both herbicides compared to the modified carbon black.

Recently, work on the adsorption of 2,4-D and MCPA from water on N-220 carbon black modified via H_2_O_2_ oxidation and deposition of aminopropyltriethoxysilane (APTES) was published [112]. The authors reported that adsorption was pH-dependent and that the experimental data best fitted pseudo-second-order and Langmuir models for kinetic and equilibrium data, respectively. The surface chemistry of the carbon blacks plays a more important role than their textural properties. 2,4-D and MCPA were favorably adsorbed on APTES-modified carbon black and were worst on oxidized carbon black.

Other types of carbon materials used in the research on the adsorption of chlorophenoxy herbicides are carbon nanotubes, both multi-walled and single-walled.

In [113,114,115], the authors describe the research on the adsorption of the herbicides 2,4-D, 2,4,5-T, and MCPA on multi-wall carbon nanotubes. MWCNTs include both commercial and laboratory-produced materials. SWCNTs were also used for 2,4-D or MCPA adsorption studies [97,115,116]. Graphene nanosheets or rGO were also used as chlorophenoxy herbicide adsorbents [97,117]. The kinetics and equilibria of adsorption have been studied in detail. 

Interesting results were presented when ordered mesoporous carbon was used as the adsorbent [118]. Mesoporous carbon C_KIT-6_ was prepared with the use of ordered mesoporous silica KIT-6 as a template. 2,4-D herbicide adsorption was tested for the original and modified (aminosilane-functionalized) samples of C_KIT-6_ carbon. The modification resulted in a significant increase in 2,4-D adsorption despite a large reduction in the surface area of the C_KIT-6_ carbon samples. 

The magnetic and graphitic carbon nanostructures from the commercial filter paper GCN-P and cotton GCN-C were prepared and tested as adsorbents for 2,4-D removal [119]. The GCN-P showed a higher surface area (182.4 m^2^ g^−1^) compared to the GCN-C (27.4 m^2^ g^−1^). The kinetic and equilibrium data were modeled with different models. It was found that the kinetics of the adsorption of 2,4-D followed the Elovich model, while the equilibrium data followed the Redlich–Peterson isotherm. The adsorption capacities for the prepared adsorbents from filter paper and cotton were about 77 and 33 mg g^−1^, respectively.

The removal of 2,4-D from water using activated carbon fiber modified by nitric acid (N-ACF) was proposed by Li et al. [120]. Results revealed that adsorption kinetics was well described by the PSO model, and the Langmuir isotherm was the best-fitting model for adsorption (q_m_ = 555.6 mg g^−1^). Thermodynamic data suggested that adsorption was a spontaneous and endothermic process.

A study by Tang et al. [121] describes the preparation of an iron oxide nanoparticles-doped ordered mesoporous carbon functionalized with carboxylate groups (Fe/OMC) via impregnation and then calcination. The prepared Fe/OMC adsorbent was used to remove 2,4-D from the aqueous solution. The effects of contact time, pH, and initial 2,4-D concentration were studied. The kinetics and isotherm study showed that the PSO kinetic and Langmuir isotherm models fit well the adsorption data.

In another paper [122], the SBA-15-ordered mesoporous silica was used as a template for the preparation of mesoporous carbon replicas. The effects of initial herbicide concentration, contact time, solution pH, and adsorbent dosage were investigated. It was found that the adsorption kinetics of 2,4-D followed the PSO model and that the Langmuir isotherm represented experimental data with the maximum adsorption capacity of 175.4 mg g^−1^.

Adsorption of 2,4-D on magnetic Fe_3_O_4_@graphene nanocomposite was reported by Liu et al. [123]. It was observed that the 2,4-D adsorption was strongly pH-dependent, and the optimal pH value was found to be 3. The kinetics and isotherm data fitted well with PSO and Langmuir models, respectively. According to Langmuir’s equation, the maximum adsorption capacity of 2,4-D was 32.3 mg g^−1^ at 30 °C and pH 3. The thermodynamic data suggested that the adsorption was an exothermic and spontaneous process.

The graphene oxide-Fe_3_O_4_ nanocomposite (GO-Fe_3_O_4_) was synthesized and examined for the removal of 2,4-D from water [124]. Adsorption kinetics and equilibrium adsorption data were fitted to PSO and Freundlich models, respectively. A similar material, 3-dimensional/graphene oxide/magnetic (3D/GO/Fe_3_O_4_), was prepared by Hajigasemkhan et al. [125] and used for the adsorption of 2,4-D from water. The effects of pH, contact time, 2,4-D initial concentration, temperature, and adsorbent dosage were investigated. Adsorption kinetics followed the PSO equation while the equilibrium data were fitted well with Langmuir isotherm.

Magnetic-activated charcoal/Fe_2_O_3_ nanocomposite was synthesized using *Spondias dulcis* leaf extract and used for the adsorption of 2,4-D from aqueous solutions [126]. Under optimum conditions, the adsorption efficiency of 98.12% was observed. Kinetics, isotherm, and thermodynamic studies demonstrated that the equilibrium data were best fitted to Langmuir isotherm, that the kinetics followed the PSO model, and that the adsorption process was spontaneous and exothermic. Langmuir’s maximum adsorption capacity was found to be 255.1 mg g^−1^.

The graphene oxide/MIL 101(Cr) (GO/MOF) nano-composite was synthesized by a simple one-pot hydrothermal method and tested as an adsorbent for 2,4-D removal from water [127]. It was found that the kinetics and isotherm data fitted well with the PSO and the Langmuir model, respectively. The adsorption capacity of 2,4-D in an aqueous solution was found to be 476.9 mg g^−1^.

Recently, Demiti et al. [128] synthesized magnetic adsorbent from activated carbon fiber and iron oxide nanoparticles (ACF-Fe_3_O_4_) for the removal of 2,4-D. The removal of 2,4-D from an aqueous solution was investigated at various physicochemical parameters, such as pH, amount of adsorbent, contact time, and temperature. The experimental data were better described by the PSO kinetic model and by the Langmuir isotherm equation. The thermodynamic parameters suggested that the process was spontaneous, exothermic, and thermodynamically favorable. The maximum adsorption capacity was 51.10 mg g^−1^ at 15 °C.

Elutrilithe (mixed alumina-silicate/carbon material) is a solid waste from coal mines [129]. The elutrilithe was treated with ZnCl_2_ in a nitrogen medium and pyrolyzed at 700, 800, and 900 °C for 1 h. After the activation process, the surface areas of the A(700), A(800), and A(900) samples were 122.4, 119.4, and 161.9 m^2^ g^−1^, respectively. These materials were tested as adsorbents for MCPA removal from water. The adsorption experiments were carried out as a function of time, initial concentration, agitation rates, and temperature. The kinetic data obtained from the adsorption experiments were fitted to the PSO model, and the Langmuir model was used to fit the equilibrium data. The maximum monolayer adsorption capacity for A(700), A(800), and A(900) was 32.1, 30.7, and 38.7 mg g^−1^, respectively.

The results of chlorophenoxy herbicides adsorption studies on all discussed carbon materials and their properties are presented in Table 4. Analysing the adsorption parameters for the activated carbons produced from solid wastes and for other carbon materials (Table 3 and Table 4, respectively), one can find the proportionality of the maximum adsorption amount to the adsorbent surface area, as in the case of commercial active carbons.

## 5. Adsorption of Chlorinated Phenoxyacetic Acids on Non-Carbonaceous Adsorbents

### 5.1. Inorganic Materials

Nowadays, adsorption is one of the most widely used methods for chlorinated phenoxyacetic acid removal from waters, and among numerous adsorbents, inorganic materials are also applied. Silica gels, activated alumina, zeolites, and molecular sieves belong to the inorganic materials that deserve special attention due to their remarkable advantages of chemical inertness, thermal and mechanical stability, high surface area, low cost, as well as the possibility of subjecting them to a regeneration process and further reuse [130,131,132].

The porous structure of some inorganic materials can be additionally controlled and adjusted for a specific pollutant at the stage of synthesis [133]. However, based on the literature, one can state that effective treatment of waters polluted with chlorophenoxy herbicides highly depends on the surface chemistry of inorganic adsorbents. It is difficult to find reports on the utilization of pristine inorganic materials in the adsorption process of chlorinated phenoxyacetic acids, and, if they are, then they are rather for comparative purposes only. Inorganic materials subjected to a modification that generally exhibit a better removal performance for this type of compound are more popular. The selection of the appropriate modification is closely related to the physicochemical properties of pristine adsorbents and pollutants.

This chapter presents research results on the purification of waters from chlorophenoxy herbicides with various inorganic materials as adsorbents in unmodified and modified forms. The modification process of inorganic materials includes simple surface functionalization, grafting, anchoring, imprinting processes, or creating a metal-organic framework. The influence of adsorbents’ properties and experimental conditions on the adsorption process along with probable pollutant-adsorbent interactions is reported.

In the paper [134], synthetic calcite, quartz, and α-alumina were used for the 2,4-D removal from aqueous solutions. The obtained results showed that these minerals in a pure form were characterized by poor adsorption capacity. It was noted that adsorption mainly depended on a type of mineral, mineral-specific surface area, and surface charge as well. Adsorption of the herbicide on the solids occurred only at the pH values where minerals’ surfaces contained positive sites. The addition of an electrolyte proved to be a factor that diminished the adsorption process as a result of weak interactions between pollutant and adsorbent. For this reason, the authors suggested that a process had nonspecific adsorption features that involved the electrostatic bonding mechanism. The anionic herbicide was attracted by the $=SiOH_{2}^{+}$, $=Ca(OH_{2}{)}^{+}$, $=AlOH_{2}^{+}$ surface sites of quartz, calcite, and α-alumina, respectively. For all systems, the adsorption data corresponding to low equilibrium concentrations were defined by linear isotherms. For higher equilibrium concentrations the data followed the Freundlich equation (2,4-D/synthetic calcite) or exhibited an S-shape isotherm tendency (2,4-D/quartz and 2,4-D/α-alumina).

Goyne et al. [135] reported results of the 2,4-D adsorption on aluminas and silicas with varying degrees of mesoporosity. They observed that the herbicide did not adsorb onto silicas, irrespective of adsorbent porosity characteristics, while adsorption on aluminas was distinctly pore-type dependent. The alumina of the highest mesoporosity adsorbed more herbicide per unit of surface area than did the nonporous and less mesoporous ones. Differences in adsorption efficiency were noted despite equivalent surface site densities of the aluminas adsorbents. The adsorption data were fitted by the Freundlich equation. A concave shape of excess isotherms reflected a contribution of lateral adsorbate/adsorbate interactions at the alumina surface. The postulated thesis was confirmed by satisfying data fitting to the Frumkin–Fowler–Guggenheim (FFG) equation. The adsorption process on aluminas was fast, and in all studied systems, equilibrium was achieved within a time of <30 min. The authors suggested that pores significantly larger (D_pore_ = 8–10 nm) than the solute molecule (effective size < 1 nm) did not contribute to diffusion-limited adsorption/desorption hysteresis. The lack of adsorption on silicas was explained by the occurrence of electrostatic repulsion, while binding of 2,4-D to aluminas was probable based on the electrostatic attraction mechanism between dissociated carboxylate groups and positively charged aluminon groups. ATR-FTIR studies excluded adsorption via ligand exchange.

The authors of [136] carried out the adsorption of 2,4-D on HY, Hβ, and HZSM-5 zeolites. The order of zeolites’ adsorption capacity was: HY > Hβ > HZSM-5. It emphasized a good correlation between zeolites’ adsorption capacity and the number of protonic sites on their surfaces. Since 2,4-D is polar in nature, greater adsorption on the HY and Hβ zeolites was observed due to their low Si/Al ratio. For all adsorption systems, a time of 30 min was enough to achieve an equilibrium state.

The research group of Ortiz Otalvaro [137] showed that a modification of ordered mesoporous silica MCM-41 with a coupling agent of 3-aminopropyltriethoxysilane (APTES) could significantly enhance the adsorption of 2,4-D. This compound does not exhibit affinity to the pure silica surface. According to the authors’ estimations, the adsorption capacity of the obtained product was about 75 times higher than for a pristine material. Its better performance was connected to a reverse charge development on the solid surface. The adsorption was strongly dependent on the pH and ionic strength values. The highest adsorption at pH = 4.5 and without electrolyte in solution was observed. Adsorption kinetics of 2,4-D was extremely fast at the initial period of the experiment (below 5 min), and the equilibrium state after 120 min was reached. Electrostatic attractions and hydrogen bonds between protonated aminopropyl groups of adsorbent and negative functional groups of adsorbate were indicated as responsible for the adsorption process. Based on reusability experiments, it was indicated that the performance of the adsorbent decreases up to 75% after four successive cycles of adsorption/desorption processes due to the limited stability of APTES on the mesoporous silica MCM-41.

Performance of modified mesoporous silicas by a grafting process with 3-aminopropyltriethoxysilane (APTES) and 3-ureidopropyltrimethoxysilane (TMSPU) for adsorption of 2,4-D was tested [133]. The results showed that the functionalization of silicas’ surface with APTES significantly improved the adsorption capacity of the as-modified adsorbents for 2,4-D. The values of this parameter estimated from the Langmuir model were close to 280 mg g^−1^ and, generally, they were ~8-fold higher compared to those for parent materials and ~4-fold higher compared to those for TMPSU-functionalized materials. Based on the kinetic experiments, it was estimated that an equilibrium state was achieved just after 90 min, and that the process ran with a pseudo-second-order model. The functionalization of silicas’ surface with TMPSU showed only a slight increase in adsorption capacity for 2,4-D compared to non-modified silica materials.

Based on a literature review of the subject, it can be found that the insertion of appropriate moieties into a solid surface translates into adsorption efficiency. Generally, more functional group density on solid surfaces means more spectacular adsorption results. This, in turn, is strictly dependent on a modification method.

Alotaibi et al. [138] demonstrated that anchoring a solid surface with polymer chains by a subsequent polymerization treatment can significantly increase the extent of the surface functionalization and improve adsorption performance. The author grafted polymer brushes (poly(2-(tert-butylamino)ethyl methacrylate)) onto the surface of mesoporous silica and applied it successfully to remove the 2,4,5-trichlorophenoxyacetic acid (2,4,5-T). The porous structure of the material along with the pH-responsive feature of the polymer chain allowed the extraction of the herbicide with steady efficiency in the pH range of 3–7. The maximum adsorption capacity of modified silica for 2,4,5-T was 290 mg g^−1^. The equilibrium and kinetic data were best described by the Freundlich isotherm and pseudo-second-order equation, respectively. It was suggested that more than one mechanism in the adsorption process was involved, indicating the electrostatic and π-π interactions.

In the work of Prado and Airol [139], it was shown that an adsorbent obtained by modification of silica gel by anchoring APTES on its surface followed by 2,4-D immobilization can be used to adsorb the same herbicide. The experimental study was conducted at the pH range of 1–7. From a series of adsorption isotherms fitted to a modified Langmuir equation, the maximum retention capacity for the organofunctionalized silica was determined as 4.67 × 10^−5^ mol g^−1^ at pH 5. The hydrogen bonds between herbicide carboxylic groups and corresponding available moieties immobilized on the silica surface were created.

Molecularly-imprinted amino-functionalized silica gel exhibited a high potential for the removal of 2,4-D [140]. The adsorbent was prepared by functionalization of its surface with APTES followed by incorporation of 2,4-D as template molecules. The last stage of adsorbent preparation included the removal of the template from the rigid silica gel—APTES networks, which led to obtaining the cross-linked product with high adsorption efficiency towards 2,4-D. In single compound adsorption experiments, the greatest loading capacity for herbicide at pH ranging of 2.5–3.4 was observed. The effect of pH involved ionic interactions between 2,4-D and adsorbent. The value of the loading capacity for an initial 2,4-D concentration of 900 mg L^−1^ was 125 mg g^−1^. The experimental results of adsorption from the binary solutions showed high selectivity of as-prepared adsorbent to the 2,4-D compared to a competing compound (2,4-dichlorophenol or 2,4-dichlorophenylacetic acid). Furthermore, the adsorption process on the imprinted functionalized silica gel was characterized by fast kinetics with a removal efficiency of 73% within 5 min. The reusability test (five extraction/stripping cycles) demonstrated good recyclability of adsorbent with a performance of 93% compared to fresh material.

Isiyaka et al. [141] proved the superiority of aluminum-based metal-organic framework MIL-53(Al) in the removal of MCPA compared to other adsorbents reported. The material was characterized by a large surface area (1104 m^2^ g^−1^) and pore volume (0.63 cm^3^ g^−1^), which contributed to high performance. The adsorption data were best fitted by the Freundlich isotherm, but using the central composite design RSM and ANN optimization models, a maximum adsorption capacity was estimated to be 231.9 mg g^−1^. The equilibrium state was reached within 25 min using merely 0.01 g L^−1^ of adsorbent. The adsorption process proceeded according to the pseudo-second-order model. The removal efficiency of the material on the level of 90% in the reusability experiments (five cycles) was also promising in the prospect of MIL-53(Al) use for water remediation.

The results of studies of kinetic and equilibrium adsorption of chlorophenoxy herbicides on selected inorganic materials are listed in Table 5.

### 5.2. Non-Conventional (Low-Cost) Adsorbents

The conventional adsorbents, e.g., activated carbons, are expensive and energy-intensive. Therefore, over the last few years, much work has been conducted to explore an alternative to expensive materials. Several kinds of materials have been used for the adsorption process to test their adsorption potential and effectiveness. Such materials either occur naturally or are considered as waste and their utilization as low-cost adsorbents for the treatment of wastewater may make them of some value, and, at the same time, contribute to waste minimization, recovery, and reuse. The main factors characterizing these materials are their affordability, local availability, and efficiencies in the removal of many inorganic and organic pollutants, and they could therefore be utilized instead of conventional but expensive adsorbents.

Based on the classification introduced by Crini et al. [142], non-conventional adsorbents can be classified into six categories: (1) natural materials; (2) agricultural wastes; (3) industrial by-products; (4) biosorbents; (5) miscellaneous adsorbents; and (6) activated carbons from solid wastes. A similar classification of low-cost materials was proposed by Ali et al. [143], who categorized these materials as (1) soil and ore materials, (2) agricultural products, (3) household wastes, (4) industrial waste, (5) sea materials, and (6) metal oxides and hydroxides.

In this paper, the classification of adsorbents used to remove herbicides from the water was based on these two articles [142,143], but some changes were introduced. Table 6, Table 7, Table 8 and Table 9 summarize the non-conventional materials and show their adsorption capacity towards the two most studied herbicides—2,4-D and MCPA.

#### 5.2.1. Natural Materials

The adsorption of phenoxy herbicides on various natural materials, including soils, clays, minerals, and rocks, has been studied by many research centers. However, in the case of soils, the aim of the research was not so much to test them as low-cost adsorbents but rather to test their adsorption capacity and in the light of the protection of groundwater against the penetration of herbicides into them. The fate and transport of the phenoxy herbicides (adsorption/desorption) in the subsurface are affected by a complex, time-dependent interplay between adsorption and mineralization processes, which are influenced by both the water and organic matter content of the soil as well as its pH. Research on the adsorption of phenoxyacetic acids concerned soils with different degrees of mineralization, different contents of organic materials, different geological origins, and different locations [50,144,145,146,147,148,149,150,151,152]. The problem of adsorption and transport of herbicides in the soil has been recently very carefully described and discussed in the review by Paszko et al. [153]. 

A very important group of natural adsorbents used in the adsorption of herbicides are clay minerals. Bakhtiary et al. [154] studied the adsorption-desorption behavior of 2,4-D on surfactant (N-cetylpyridinium) modified bentonite and zeolite. Adsorption of the herbicide on the modified bentonites and zeolites was much higher than those of unmodified materials. The 2,4-D adsorption capacity (q_max_) on raw bentonite was 6.5 µmol g^−1^ while on the NCP-modified bentonite increased to 171.1 µmol g^−1^. The authors observed that the 2,4-D adsorption capacity of the organo-minerals increased with increasing surfactant loading. 

The performance of organophilic clays obtained from the chemical modification of sodium bentonite clay for adsorption of 2,4-D was tested [155]. Adsorption was pH-dependent, and the best adsorption efficiency was within the pH range of 3–8. Adsorption kinetics followed the PSO model while Dubinin–Radushkevich was the model that best fitted the experimental isotherm data. At 25 °C, the maximum adsorption capacity was 50.36 mg g^−1^. The thermodynamic study suggested physical adsorption occurring spontaneously in the system.

In another work [156], the authors investigated the use of two raw bentonites (Wyoming and Clair T) as low-cost adsorbents for the removal of phenol, bisphenol A, ibuprofen, and 2,4-D from aqueous solutions. The results revealed that the adsorption was strongly pH-dependent and decreased with an increase in the initial pH of the solution. Adsorption kinetics was described using the PFO, PSO, Elovich, and intra-particle diffusion models, while the equilibrium adsorption data were modeled with Langmuir, Freundlich, and Langmuir–Freundlich isotherm equations. The experimental data followed the pseudo-second-order kinetic model and the Langmuir–Freundlich isotherm. The adsorption capacity of the 2,4-D was 1.19 mg g^−1^ and 1.79 mg g^−1^ on the Wyoming and Clair T bentonite, respectively.

Cationic surfactant hexadecyltrimethylammonium bromide (HDTMA) modified bentonite was tested as an adsorbent for the removal of 2,4-D, phenol, and dichromate ions from aqueous solutions [157]. The Langmuir isotherm gave a satisfactory fit to the equilibrium adsorption data for 2,4-D, and the adsorption capacity was found to be 47.2 mg/g.

Inorganic-organic bentonites prepared by intercalation of poly(hydroxo aluminum) or poly(hydroxo iron) cations followed by intercalation of cetyltrimethylammonium ions were used as adsorbents for 2,4-D and acetochlor [158]. Adsorption and desorption studies as well as the influence of pH on the adsorption of 2,4-D were carried out. The authors concluded that the pillared bentonites with subsequent adsorption of cetyltrimethylammonium ions showed enhanced 2,4-D adsorption in comparison with organo-bentonites. 

Commercial organophilic type bentonite clay (Spectrogel) was used in adsorption studies for the aqueous phase of a few pesticides, including 2,4-D [159]. The kinetic study showed that the 2,4-D adsorption was limited by intraparticle diffusion and external mass transfer steps, and the Elovich model was the most predictive of the experimental data. The adsorption equilibria were evaluated by Langmuir, Freundlich, and Temkin isotherm models, and the Langmuir model had the best adjustment to experimental data for 2,4-D. The maximum adsorption capacity in the monolayer was found to be 6.45 mg/g.

Papers by Durán et al. [160] and Bueno et al. [161] have reported the removal of pesticides, including terbuthylazine, tebuconazole, and MCPA, from aqueous solutions using raw bentonite, bentonite without carbonates, bentonite modified with hexadecyltrimethylammonium (Bent-HDTMA), and with ferric cation (Bent-Fe), as well as chitosan-modified bentonite. The results showed that the modification of the bentonite improved its adsorption capacity when compared to unmodified bentonite, although the removal efficiency depended on the modifier cation. Both the Fe(III) and HDTMA were appropriate modifiers for this purpose, but Bent-HDTMA showed better adsorption capacity for the MCPA in comparison to Bent-Fe. The bentonites modified with ferric cation and with HDTMA exhibited better adsorption behavior (40–100%) compared to chitosan-modified bentonite. Bent-HDTMA was the best adsorbent for MCPA, with an adsorption efficiency of up to 95%.

The adsorption capacities of bentonite (DB) and sepiolite (DS) modified by dodecylammonium cation for phenoxy alkanoic acid herbicides including 2,4-D, MCPA, 2,4-DP ((RS)-2-(2,4-dichlorophenoxy)propionic acid), 2,4-DB (4-(2,4-dichlorophenoxy)butyric acid), and 2,4,5-T ((2,4,5-trichlorophenoxy)acetic acid) were investigated [162]. Results showed that the Langmuir isotherm gave a poor fit for both adsorbents while there was a good correlation for the Freundlich equation. Adsorption of the studied herbicides onto DB and DS decreased in the order of 2,4-DB > 2,4,5-T > 2,4-DP > 2,4-D > MCPA. The modified sepiolite showed higher adsorption capacity than the modified bentonite for all of the herbicides.

Another paper by these authors [163] concerned the adsorption of 2,4-D on the dodecylammonium sepiolite (DAS) as a function of solution concentration and temperature. The Freundlich and Dubinin–Radushkevic isotherm equations were used for the description of the adsorption equilibrium of 2,4-D onto DAS depending on temperature. The adsorption was well described by both isotherm models at all of the temperatures studied. Obtained thermodynamic constants suggested the exothermic nature of the adsorption process.

Adsorption of four pesticides, including atrazine, diuron, paraquat, and 2,4-D, on bentonites was investigated [164]. Bentonite was modified with 0.5 M HCl (BA_0.5_), calcined at 500 °C (BC_500_), and combined acid and heat treatments. The adsorption tests evaluated the influence of the pH of the solution, temperature, and contact time on pesticide removal. The results showed that the adsorption process was a good fit for Langmuir isotherm and PSO kinetics models. Thermodynamic studies suggest a spontaneous and endothermic process.

Sepiolite nanofibers modified by N-cetylpyridinium (NCP) cations were characterized and used for the adsorption of 2,4-D from water solution [165]. The adsorption capacity of sepiolite for 2,4-D was significantly improved after NCP modification and increased with enhancing the surfactant loading. A maximum adsorption capacity of 34.46 µmol g^−1^ was determined according to the Langmuir isotherm.

The removal of 2,4-D from aqueous solution using raw and surfactant (hexadecyltrimethylammonium and dioctadecyldimethylammonium) modified minerals, including clinoptilolite, zeolite Y, bentonite, and montmorillonite, was investigated by Bhardwaj et al. [166]. In that investigation, adsorption of 2,4-D with a variation of surfactant loading, adsorbent dosage, pH of the solution, equilibration time, and temperature was examined. The equilibrium data were fitted to the Langmuir adsorption isotherm, and the maximum monolayer 2,4-D adsorption capacity values were obtained. The results showed that surface-modified minerals with HDTMA and DODMA exhibited the highest adsorption capacity compared to the raw, non-modified materials.

The elimination of 2,4-D and paraquat from an aqueous solution via adsorption on raw and modified zeolites was investigated by Pukcothanung and collaborators [167]. The surface of zeolite Y was modified using hexadecyltrimethylammonium chloride (HDTMA) and sodium dodecyl sulfate (SDS) surfactants. The equilibrium adsorption data were fitted well using the Langmuir isotherm model and the adsorption kinetics followed a PSO model. The results also showed that solution pH played an important role in the removal of both pesticides and that the greatest adsorption value for 2,4-D occurred at pH 3. Under these conditions (at a pH of 3.0), the maximum adsorption capacity of the unmodified zeolite was greater than that of the modified zeolites. The Langmuir’s adsorption capacity for 2,4-D was 175.44 mg g^−1^ for unmodified zeolite Y, 82.64 mg g^−1^ for HDTMA-modified and 120.48 mg g^−1^ for SDS-modified zeolite, respectively. However, in the pH range from 4 to 8, the surfactant-modified zeolites were more effective.

The HDTMA was also used as a modifier of Arizona montmorillonites [168]. Surfactant-treated minerals were examined for the removal of 2,4-D from water. 

In another paper, a modified montmorillonite nanoclay of commercial-grade (Cloisite 30B), which is modified with methyl tallow bis(2-hydroxyethyl) quaternary ammonium salt, was tested as an adsorbent for removal of 2,4-D from water [169]. Adsorption isotherms and kinetic studies, conducted under controlled temperature and pH conditions, were investigated. The Langmuir, Freundlich, and Dubinin–Radushkevich, as well as the pseudo-first-order, pseudo-second-order, and intraparticle diffusion models, were used for the description of the experimental data. The Freundlich and the PSO models were the best-fitted models. The highest experimental adsorption capacity (at pH = 2) was found to be 282.87 mg g^−1^.

Untreated and decylammonium-treated clays, montmorillonite (C10M) and vermiculite (C10V), were tested for removal of 2,4-D from aqueous solutions [170]. The adsorption of 2,4-D was much greater for C10V than for C10M. Herbicide adsorption at various pH values indicated that the undissociated form was preferentially adsorbed on C10M, whereas the anionic form was adsorbed by C10V. 

Celis et al. [171] prepared a series of modified Na-rich Wyoming and Ca-rich Arizona montmorillonites to remove terbuthylazine, diuron, and MCPA from aqueous solutions. The organic cations used for the modification of the clay minerals were L-carnitine, spermine, phenyltrimethylammonium, hexadecyltrimethylammonium, hexadimethrine, and tyramine. Modification of the montmorillonites with these organic cations rendered organoclays with excellent affinities for all three herbicides. Results showed that the modified montmorillonites removed more than 95% of the herbicide initially present in the aqueous solution, whereas the unmodified clays removed less than 15%. 

Adsorption of MCPA on Argentine montmorillonite (MMT) and its organo-montmorillonite product treated with different dodecyl trimethyl ammonium loading was investigated by Santiago et al. [172]. Montmorillonite (MMT2) modified with the organic cation through ion exchange reaction under the most drastic conditions (200% cation exchange capacity) showed the best adsorption capacity, several times greater than the raw, unmodified MMT. Adsorption isotherms were fitted by several models, including Langmuir, Freundlich, Dubinin–Radushkevich, and Toth. The adsorption process satisfactorily fits with the Langmuir model, and Langmuir’s q_m_ values were found to be 0.034 and 0.284 mmol g^−1^ for the MMT and MMT2, respectively.

The adsorption capacity of montmorillonite modified by N,N′-didodecyl-N,N′-tetramethylethanediammonium (DEDMA) cations towards phenoxyalkanoic herbicides (MCPA, 2,4-D, 2,4-DB, and 2,4,5-T) was also studied [173]. Adsorption isotherms were modeled according to Freundlich and Dubinin–Radushkevic isotherms, and the adsorption process was described better using the D-R equation. The adsorption of the herbicides on DEDMA-montmorillonite increased in the order of MCPA < 2,4-D < 2,4-DB < 2,4,5-T. The D–R q_m_ parameters obtained for DEDMA-montmorillonite were 0.535, 0.548, 0.549, and 0.552 mmol g^−1^ for MCPA, 2,4-D, 2,4-DB, and 2,4,5-T, respectively.

Australian palygorskite modified with octadecyl trimethylammonium bromide (OP2CEC) and dioctadecyl dimethylammonium bromide (DP2CEC) ions were tested as low-cost adsorbents for removal of 2,4-D from water [174]. The results revealed that the unmodified palygorskite adsorbed negligible amounts of 2,4-D and the adsorption capacity was significantly improved by modifying palygorskite with the surfactants. The Langmuir’s maximum adsorption of 2,4-D on OP2CEC and DP2CEC was 42.02 and 25.77 mg g^−1^.

Pavlovic et al. [175] studied the adsorption capacity of hydrotalcite Mg_3_Al(OH)_82_CO_3_·*n*H_2_O and its calcined product (HT500) as potential adsorbents of three acidic pesticides: 2,4-D, clopyralid, and picloram in water. Adsorption isotherms, adsorption kinetics, as well as the effect of solution pH, were studied, and the results showed that the intensity of the adsorption was related to the acidity of the pesticides. Adsorption isotherms were described using the Langmuir equation, and the maximum adsorption capacity of 2,4-D on HT500 was 0.813 mmol g^−1^.

In other studies, bituminous shale was used for 2,4-D [176] and MCPA [177] removal from water. The effect of the experimental parameters, including temperature, pH value, contact time, and adsorbate concentration using the batch technique, was investigated. Adsorption capacities of the bituminous shale increased with increasing temperature and decreasing pH. Adsorption isotherm was described using Langmuir, Freundlich, Dubinin–Radushkevich, and Temkin equations. The maximum adsorption capacity based on the Langmuir model was found to be 5.45 µmol g^−1^ for 2,4-D and 5.09 µmol g^−1^ for MCPA.

The adsorption behavior of the phenoxyacetic acid herbicides (phenoxyacetic acid (PAA), 2,4-D, MCPA, and 2,4,5-T) on goethite was studied by Kavanagh et al. [178]. The adsorption of the herbicides on goethite increased in the order of PAA < 2,4-D < MCPA < 2,4,5-T. Adsorption of MCPA on goethite [179] as well as on goethite and humic acid-coated goethite [180] was also investigated by other authors.

In a recently published study, raw lignite was tested as a low-cost adsorbent for the removal of phenoxyacetic acid (PAA), 4-CPA, 2,4-D, and MCPA from aqueous media [181]. The adsorbent was studied without any pretreatment in batch experiments. Results demonstrated that adsorption was pH- and ionic strength-dependent. The adsorption kinetics and adsorption equilibrium data were well-fitted with the PSO and Freundlich models, respectively. The adsorption capacities for PAA, 4-CPA, MCPA, and 2,4-D were 3.123, 5.056, 7.431, and 7.783 mg g^−1^, respectively.

Table 6 summarizes the adsorption capacity of 2,4-D and/or MCPA onto various natural materials.

**Table 6:** The results of chlorophenoxy herbicide adsorption studies on natural materials.

Adsorbent	BET m^2^ g^−1^	Adsorption Capacity (q_m_) mg g^−1^		Isotherm Model	Kinetic Model	Ref.
		2,4-D	MCPA			
Andisol soil	-	0.0095	-	-	PFO, PSO, E, W-M	[151]
bentonite	-	0.014	-	L, F	-	[154]
N-cetylpyridinium modified bentonite	-	0.378	-	L, F	-	[154]
N-cetylpyridinium modified zeolite	-	0.130	-	L, F	-	[154]
Modified bentonite	-	136.1	-	L, F, D-R	PFO, PSO, B, E, W-M	[155]
Raw bentonite (Wyoming)	10.2	1.19	-	L, F, L-F	PFO, PSO, E	[156]
Raw bentonite (Clair T)	79.3	1.79	-	L, F, L-F	PFO, PSO, E	[156]
HDTMA modified bentonite	2.0	47.2	-	L	-	[157]
Commercial bentonite clay	-	6.45	-	L, F, Te	PFO, PSO, E, W-M	[159]
Dodecylammonium bentonite	-	0.0003	0.0001	L, F, H	-	[162]
Dodecylammonium sepiolite	-	0.046	0.0008	L, F, H	-	[162]
Non-modified bentonite	31.76	0.32	-	L, F, Te, D-R	PFO, PSO, E, W-M	[164]
Acid-treated bentonite BA_0.5_	77.12	0.31	-	L, F, Te	PFO, PSO, W-M	[164]
Heat-treated bentonite BC_500_	32.19	0.28	-	L, F	PFO, PSO	[164]
N-cetylpyridinium modified sepiolite	-	0.076	-	L, F	-	[165]
Clinoptilolite	-	42.02	-	L, F	-	[166]
Clinoptilolite HLC	-	94.34	-	L, F	-	[166]
Clinoptilolite DLC	-	75.19	-	L, F	-	[166]
Zeolite Y	-	51.81	-	L, F	-	[166]
Zeolite Y HLY	-	116.3	-	L, F	-	[166]
Zeolite Y DLY	-	120.5	-	L, F	-	[166]
Bentonite	-	121.9	-	L, F	-	[166]
Bentonite HLB	-	129.9	-	L, F	-	[166]
Bentonite DLB	-	133.3	-	L, F	-	[166]
Montmorillonite	-	78.13	-	L, F	-	[166]
Montmorillonite HLM	-	158.7	-	L, F	-	[166]
Montmorillonite DLM	-	161.3	-	L, F	-	[166]
Zeolite Y	744	175.4	-	L, F	PFO, PSO	[167]
HDTMA-modified zeolite	299	82.64	-	L, F	PFO, PSO	[167]
SDS-modified zeolite	548	120.5	-	L, F	PFO, PSO	[167]
Montmorillonite (Cloisite 30B)	-	282.9	-	L, F, D-R	PFO, PSO, W-M	[168]
Argentine montmorillonite	573	-	0.0069	L, F	-	[172]
DDTMA treated montmorillonite	108	-	0.0570	L, F	-	[172]
OP2CEC organo-palygorskite	33.2	42.02	-	L	-	[174]
DP2CEC organo-palygorskite	25.6	25.77	-	L	-	[174]
Calcined hydrotalcite	-	0.0018	-	L	-	[175]
Bituminous shale	11.0	0.0114	-	L, F, D-R	M-P	[176]
Bituminous shale	11.0	-	0.0101	L, F, D-R, Te	M-P	[177]
Lignite	0.91	7.783	7.431	L, F, L-F	PFO, PSO, B, W-M	[181]

#### 5.2.2. Natural Biosorbents

Using natural biosorbents to remove various water contaminants, including phenoxy herbicides by adsorption, has been widely studied. Examples of such natural adsorbents are chitin and chitosan, which are some of the most abundant natural, non-toxic, and biodegradable biopolymers. Adsorption of 2,4-D on chitin and chitosan [182] from the water was studied. The results showed that the adsorption was dependent on the solution pH (maximum at pH 3.7), the initial concentration of the 2,4-D, and the herbicide-adsorbent contact time. The adsorption was described by Langmuir and Freundlich isotherm models. The adsorption capacity calculated from the Langmuir equation was 6.08 mg g^−1^ for chitin and 11.2 mg g^−1^ for chitosan, respectively.

The polyethyleneimine-modified fungal biomass of *Penicillium chrysogenum* was prepared and used for the removal of the anionic pentachlorophenol and 2,4-D from aqueous solutions [183]. The adsorption was performed at different pH values and initial herbicide concentrations. Both the adsorption kinetics and adsorption isotherms were investigated. The adsorption was pH-dependent. The adsorption kinetics and isotherms were well described by the pseudo-second-order and Langmuir models, respectively, indicating the possible chemisorption and monolayer adsorption. The modified biosorbent was much more effective in removing 2,4-D than the pristine fungal biomass. Langmuir’s maximum adsorption capacity of 2,4-D was found to be 0.40 mmol g^−1^ for the pristine fungal biomass and 1.22 mmol g^−1^ for the aminated biosorbent, respectively.

Aswani and Kumar [184] prepared a novel biosorbent from the water hyacinth root powder. The biomass was modified with acid, thermal, ultrasound, thermal-acid, and ultrasound-acid treatments. The adsorption behaviors, including adsorption kinetics, adsorption isotherms, biosorbent dosage, solution pH, and initial herbicide concentration, were investigated. Results showed that the ultrasound-acid-modified biosorbent exhibited the best adsorption properties towards 2,4-D, with Langmuirs’s maximum adsorption capacity of 40.0 mg g^−1^. The adsorption kinetic data were well-fitted to the PSO kinetic model.

A *Physalis peruvian* chalice treated with strong acid was tested as a low-cost biosorbent to remove 2,4-D from water [185]. The adsorption behaviors, including adsorption kinetics, adsorption isotherms, thermodynamics, the effect of adsorbent dosage, and solution pH, on the adsorption characteristics were investigated in detail. The adsorption rate was evaluated by PFO, PSO, Elovich, and n-order models. For the adsorption equilibrium data, the Langmuir, Freundlich, and Toth isotherm models were tested. In this work, adsorption of 2,4-D was favored at pH = 2 and with a dosage of 0.8 g L^−1^. The PSO model was the best to represent kinetic data while the isothermal experiments were well represented by the Langmuir and Toth isotherms, reaching a maximum capacity of 244.2 mg g^−1^ for the Langmuir model and 320 mg g^−1^ for the Toth model, respectively. The authors concluded that the *Physalis peruviana* chalice treated with strong acid presented great potential as an alternative material for the removal of 2,4-D herbicide from the water.

Ultrasound-acid-modified *Merremia vitifolia* biomass was prepared and tested as a biosorbent for the removal of 2,4-D from the liquid phase [186]. The operational conditions, such as stirring speed, contact time, biosorbent dosage, solution pH, and initial herbicide concentration, were studied. The kinetics, equilibrium, and mechanism of biosorption were also investigated. The kinetic data of the biosorption was fitted using PFO and PSO kinetic equations, while the mechanism of biosorption was investigated using Weber–Morris and Boyd models. The Langmuir, Freundlich, Temkin, and Dubinin–Radushkevich isotherm models were tested for a description of 2,4-D adsorption at equilibrium. Adsorption kinetics was best described by the PFO model and the equilibrium biosorption data followed the Langmuir isotherm model with a maximum adsorption capacity of 66.93 mg g^−1^. The thermodynamic parameters calculated from the equilibrium data indicated that the adsorption process was spontaneous, favorable, and exothermic.

In a subsequent article, the authors examined the adsorption of 2,4-D on acid-thermally modified *Merremia vitifolia* biosorbent [187]. Adsorption kinetic data were best fitted by the PSO model, whereas the equilibrium data were best fitted to Langmuir adsorption isotherm. The maximum monolayer adsorption capacity was 181.4 mg g^−1^.

Untreated jute (*Chorcorus olitorius*) powder as well as neem oil-phenolic resin-treated jute were tested as biosorbents for the removal of 2,4-D from water [188]. The adsorption process was found to be spontaneous and effective over a pH range between 2 and 8. The PFO, PSO, as well as the Langmuir and Freundlich models, were used to describe the biosorption process. Modified jute showed higher herbicide removal efficiency than the untreated jute; the maximum 2,4-D removal capacity of treated and non-treated jute was found to be 16.1 mg g^−1^ and 38.5 mg g^−1^, respectively. 

The use of bacterial biomass as a biosorbent was reported by Ozdemir et al. [189] and Kumar et al. [190]. In the first paper [189], the adsorption of three herbicides including 2,4-D, 2,4-DP (2,4-dichlorophenoxy propanoic acid) and 2,4-DB (2,4-dichlorophenoxy butyric acid) on thermophilic bacteria *Anoxybacillus flavithermus* biomass was investigated. Optimal values of several batch biosorption parameters, namely, contact time, pH of the solution, amount of biomass, and initial herbicides concentrations, were studied and were found as 60 min of contact time, pH = 4.0, 50 mg of bacteria, and initial adsorbates concentrations of 50 mg L^−1^. Biosorption of all the herbicides onto *A. flavithermus* biomass followed the PSO rate kinetics. The equilibrium adsorption data were fitted in both the Langmuir and Freundlich adsorption models. The maximum monolayer adsorption capacity was reported as 24.15 mg g^−1^ for 2,4-D, 13.77 mg/g for 2,4-DB, and 13.71 mg g^−1^ for 2,4-DP, respectively. The second paper [190] described the adsorption of 2,4-D and paraquat by an *Oscillatoria* sp.-dominated cyanobacterial mat. Results showed that adsorption of 2,4-D was strongly pH-dependent and was at its maximum at pH 2. The Freundlich and Langmuir equations were used to model the adsorption isotherm data, while the kinetic data were fitted to PFO and PSO models. The Langmuir model more correctly described the 2,4-D adsorption surface, while 2,4-D adsorption kinetics was well defined by both the PFO and PSO models. The maximum monolayer adsorption capacity of the mat biomass for 2,4-D and paraquat was 0.04 mmol g^−1^ and 0.13 mmol g^−1^, respectively.

Adsorption kinetics, isotherm, and thermodynamics of 2,4-D on raw and boiling-treated sterile bracts of *Araucaria angustifolia* as low-cost, natural adsorbents were also studied [191]. The adsorption processes can be optimized by controlling the bract granulometry, contact time, solution pH, adsorbent dose, and initial 2,4-D concentration. Results revealed that the maximum removal efficiency of 2,4-D was found with 720 min of contact, 5.0 g of bract, pH = 2.0, and at room temperature. The PFO and PSO kinetic models, as well as Langmuir, Freundlich, Redlich–Peterson, and Sips isotherm models, were tested. The best kinetic and isotherm fits were found with the PSO and Freundlich models, respectively. The adsorption capacity of 2,4-D on raw and boiling-treated sterile bracts of *Araucaria angustifolia* was reported as 109.81 mg g^−1^ and 126.27 mg g^−1^, respectively.

Alam et al. [192] tested the usefulness of five low-cost materials selected from biological, organic, and inorganic sources to remove the 2,4-D and atrazine from water. As adsorbents they chose macrofungi *Sajor caju* and *Florida*, wood charcoal, bottom ash collected from a local power plant, and rubber granules derived from a recycled waste tire. The Langmuir, Freundlich, Brunauer–Emmett–Teller, and Lopez–Gonzalez models were used to determine the adsorption capacity of these materials. Freundlich’s model fitted well with experimental data for most of the cases. The adsorption capacity (q_max_) for 2,4-D was 0.810 mg/g^−1^ for *Sajor caju* biomass, 0.830 mg g^−1^ for wood charcoal, 0.834 mg g^−1^ for bottom ash, 0.880 mg g^−1^ for fungi *Florida* biomass, and 0.900 mg g^−1^ for rubber granules, respectively.

Table 7 presents the compilation of various biomass-based adsorbents and their maximum adsorption capacity toward 2,4-D and/or MCPA removal.

**Table 7:** The results of chlorophenoxy herbicides adsorption studies on various natural biosorbents.

Adsorbent	BET m^2^ g^−1^	Adsorption Capacity (q_m_) mg g^−1^		Isotherm Model	Kinetic Model	Ref.
		2,4-D	MCPA			
Chitin	-	6.08	-	L, F	-	[182]
Chitosan	-	11.20	-	L, F	-	[182]
Aminated *Penicillium chrysogenum* biomass	-	0.270	-	L	PFO, PSO, W-M	[183]
Pristine *Penicillium chrysogenum* biomass	-	0.088	-	L	PFO, PSO, W-M	[183]
Water hyacinth based biosorbent	-	40.0	-	L	PFO, PSO, W-M	[184]
Modified *Physalis peruvian* chalice	-	244.2	-	L, F, To	PFO, PSO, W-M	[185]
Modified *Merremia vitifolia* biomass	-	66.93	-	L, F, Te, D-R	PFO, PSO, W-M, B	[186]
Modified *Merremia vitifolia* biomass	196.2	181.4	-	L, F, Te, D-R	PFO, PSO, W-M, B	[187]
Untreated jute powder	-	16.1	-	L, F	PFO, PSO	[188]
Modified jute powder	-	38.5	-	L, F	PFO, PSO	[188]
*Anoxybacillus flavithermus* biomass	-	24.15	-	L, F	PFO, PSO	[189]
*Oscillatoria sp*.- cyanobacterial mat	-	0.088	-	L, F	-	[190]
Raw sterile bracts of *Araucaria angustifolia*	-	109.8	-	L, F, R-P, S	PFO, PSO	[191]
Boiling-treated sterile bracts of *Araucaria angustifolia*	-	126.3	-	L, F, R-P, S	PFO, PSO	[191]
Macro fungi *Sajor caju*	-	0.810	-	L, F	-	[192]
Macro fungi *Florida*	-	0.880	-	L, F	-	[192]

#### 5.2.3. Industrial By-Products

Industrial waste, even more so than agricultural waste, has little or no economic value and often presents a disposal problem. Therefore, in recent years, many researchers have valorized these waste materials as adsorbents in water treatment. Table 5 presents the compilation of various industrial by-product adsorbents in the removal of phenoxy herbicides.

Alam et al. [192,193,194] used waste tire rubber granules to adsorb 2,4-D in a liquid phase. The equilibrium time was found to be 120 min and the adsorption followed first-order reversible kinetics. Thermodynamic data showed that the adsorption was spontaneous.

Deokar et al. investigated the potential of bagasse fly ash, a common waste generated in sugar-power industries, for 2,4-D [195] and MCPA [196] removal from aqueous solution using batch and continuous packed-bed adsorption. In the batch process, the effects of adsorbent dosage and particle size, initial herbicide concentration, time, pH, and temperature on adsorption were studied. The Langmuir isotherm model better describes the adsorption of 2,4-D, while the adsorption of MCPA was successfully fitted to the Langmuir and Temkin adsorption isotherms.

The adsorption of 2,4-D on fly ash from an electrical power plant fueled by brown coal was studied [197]. The effects of adsorbent dose, contact time, pH, ionic strength, and temperature on herbicide adsorption were investigated. Adsorption kinetic data were analyzed using PFO, PSO, and Weber–Morris models. Adsorption equilibrium was achieved after approximately 60 min; adsorption kinetics was better represented by the PSO equation. The Langmuir isotherm model fits the equilibrium data better than the Freundlich isotherm model. Thermodynamic data indicated that the adsorption process was spontaneous and endothermic.

Adsorption of 2,4-D and carbofuran from aqueous solution on fertilizer industry waste (carbon slurry) and steel industry wastes (blast furnace slag, dust, and sludge) was investigated [105]. The adsorption experiments were performed with the adsorbents under different experimental conditions (solution pH, herbicide-adsorbent contact time, adsorbent particle size, and temperature), followed by kinetic, equilibrium, and thermodynamic studies. Adsorption was found to be in decreasing order: carbon slurry (212 mg g^−1^), blast furnace sludge (30 mg g^−1^), dust 21 mg g^−1^, and slag (negligible), respectively. 

The silica gel waste modified with cetyltrimethylammonium bromide was prepared for the applications of 2,4-D removal from water [198]. Kinetics and isotherm as well as the effects of solution pH and electrolyte content were investigated. The presence of electrolyte and solution pH was found to affect the adsorption process—adsorption efficiency decreased in the presence of electrolytes and at higher pH. Equilibrium adsorption data were applied to four different adsorption models, namely, Langmuir, Freundlich, Temkin, and Redlich–Peterson. The adsorption of 2,4-D on the surfactant-modified silica gel waste followed the R-P isotherm model best compared to other isotherm models. The maximum adsorption capacity obtained from the Langmuir isotherm was 33.9 mg g^−1^ at 30 °C.

The removal of MCPA from water by activated spent bleaching earth was studied as a function of time, initial concentration, adsorbent concentration, and temperature [199]. Adsorption equilibrium was achieved within 15 min and the adsorption kinetics followed the PFO equation. The Langmuir and Freundlich isotherms were fitted by the adsorption data. The equilibrium adsorption capacity was found to be 0.08 mmol g^−1^ from the Langmuir equation.

The results of chlorophenoxy herbicide adsorption investigations on industrial by-products are presented in Table 8.

**Table 8:** The results of chlorophenoxy herbicide adsorption studies on industrial by-products.

Adsorbent	BET m^2^ g^−1^	Adsorption Capacity (q_m_) mg g^−1^		Isotherm Model	Kinetic Model	Ref.
		2,4-D	MCPA			
Rubber granules	-	0.900	-	L, F	-	[192]
Bagasse fly ash	52	5.63	-	L, F, Te	-	[195]
Lignite fly ash	12.9	0.004	-	L, F	PFO, PSO, W-M	[197]
Blast furnace sludge	28	30.0	-	L, F	-	[105]
Blast furnace dust	13	21.0	-	L, F	-	[105]
Surfactant modified silica gel waste	194	33.9	-	L, F, Te, R-P	PFO, PSO	[198]
Activated spent bleaching earth	198	-	0.016	L, F	PFO, W-M	[199]

#### 5.2.4. Metal Oxides and Hydroxides

Due to their unique physicochemical properties (numerous surface active sites, high chemical stability, adjustable shape, and size), metal oxides and hydroxides are of great interest as adsorbents [200]. 

Adsorption of 2,4-D from the aqueous phase by in situ generated metal hydroxides using sacrificial anodes was reported by Kamaraj et al. [201]. Different experimental parameters such as the effect of anode materials, 2,4-D initial concentration, temperature, pH, and current density were investigated. The equilibrium adsorption was described by the Langmuir and Freundlich models, while adsorption kinetics was analyzed using PFO and PSO kinetic models. The results revealed that the 2,4-D removal efficiency of 91.0% was achieved with iron as an anode at a current density of 0.10 A dm^−2^ and pH of 7.0. Under these conditions, Langmuir’s adsorption capacity was 45.45 mg g^−1^.

In other papers, the authors reported the removal of MCPA using metal oxides such as Al_2_O_3_, TiO_2,_ and ZnO [115] and Al_2_O_3_ and Fe_2_O_3_ [202]. The specific surface areas of the oxides were 188–195 m^2^ g^−1^ for Al_2_O_3_, 325 m^2^ g^−1^ for TiO_2_, 53 m^2^ g^−1^ for ZnO, and 10,653 m^2^ g^−1^ for Fe_2_O_3_. Effects of pH, contact time, initial concentration, and adsorbent dosage on the adsorption of the herbicide were investigated. The adsorption equilibrium data were successfully fitted to the Freundlich adsorption isotherm.

Adsorption of MCPA from aqueous solutions onto MgAl-layered double hydroxides LDHs was also investigated [203]. Results revealed that the adsorption was caused by an anion exchange mechanism and depended on the pH of the solution and the charge density of the layers. The adsorption isotherms were well described by the Freundlich model. The use of calcinated Mg-Al-CO_3_-LDH as adsorbents for herbicides has also been described by Cardoso and Valim [204]. 

Nejati et al. reported the adsorption of 2,4-D [205] and MCPA [206] on Cu-Fe-NO_3_ layered double hydroxide. Adsorption of MCPA on the nanoparticles of Cu-Fe-NO3-LDH was studied as a function of pH, contact time, and temperature. The adsorption kinetics was tested for PFO, PSO, Elovich, and intra-particle diffusion kinetic models while the equilibrium adsorption was described by Langmuir and Freundlich isotherm equations. The Langmuir isotherm was slightly better fitted to the experimental data rather than that of Freundlich, with a maximum adsorption capacity of 1428 mg g^−1^ for 2,4-D and 800 mg g^−1^ for MCPA, respectively.

Legrouri et al. [207] investigated the adsorption of 2,4-D on Zn-Al-Cl-LDH. The process was well described by the Langmuir isotherm with a maximum adsorption capacity of 5.24 mmol g^−1^. Calisto et al. investigated the removal of 2,4-D by Co-Al-Cl-LDH [208]. The kinetics of the 2,4-D adsorption was best described by the PSO kinetic model, and the adsorption equilibrium data were successfully fitted to the Freundlich adsorption isotherm. The adsorption capacity of the herbicide on Zn-Al-Cl-LDH at 25 °C was reported as 30.12 mg g^−1^.

Table 9 summarizes the application of metal oxides and hydroxides to phenoxy herbicides removal.

**Table 9:** The results of chlorophenoxy herbicide adsorption studies on metal oxides and hydroxides.

Adsorbent	BET m^2^ g^−1^	Adsorption Capacity (q_m_) mg g^−1^		Isotherm Model	Kinetic Model	Ref.
		2,4-D	MCPA			
In situ-generated metal hydroxides	-	45.4	-	L, F	PFO, PSO	[201]
Calcinated Mg-Al-CO3-LDH	62	1.111	0.938	L	-	[204]
Al_2_O_3_	195	-	13.64	F	PFO	[202]
Fe_2_O	106	-	11.84	F	PFO	[202]
Cu-Fe-NO3-LDH	-	1428	-	L, F	PFO, PSO, E, W-M	[205]
Cu-Fe-NO3-LDH	-	-	800	L, F	PFO, PSO, E, W-M	[206]
Zn-Al-Cl-LDH	-	1.158	-	L	-	[207]
Co-Al-Cl-LDH	44	30.1	-	L, F	PFO, PSO, W-M	[208]

### 5.3. Other Materials

This chapter presents various unconventional, often quite sophisticated, adsorbents that are difficult to classify in the above sections.

A magnetite-graphene oxide-layered double hydroxide composite (MGL) was synthesized, characterized, and examined as a potential adsorbent for the removal of 2,4-D from an aqueous solution [209]. Results revealed that adsorption efficiency decreased with an increase in solution pH. Adsorption was well described by both the Langmuir and Freundlich isotherms.

A composite material consisting of red mud and carbon spheres (red mud@C composite) was prepared by Kazak et al. [210] to remove 2,4-D from aqueous media. It was found that the PSO kinetic model and the Freundlich isotherm model fitted well with the adsorption behavior.

Fe-crosslinked chitosan complex was prepared in another work [211] as an adsorbent for the removal of 2,4-D from an aqueous solution. The effect of the experimental parameters, including adsorbent mass, pH value, coexisting ions, contact time, and initial concentration of 2,4-D on the adsorption capacity, was investigated. The kinetic process was well described by the PSO model, and the adsorption equilibrium data were better fitted by Langmuir isotherm. The maximum adsorption capacity of the Fe-crosslinked chitosan complex for the 2,4-D was 473 mg g^−1^.

In a study by Vieira et al. [212], the chitosan-based hydrogel and chitosan/magnetite-based composite hydrogel were prepared and used for 2,4-D removal. The batch experiments indicated that the best adsorption conditions were pH 2, an initial 2,4-D concentration of 50 mg L^−1^, and a temperature of 40 °C. Adsorption kinetics was investigated by applying PFO, PSO, and Elovich kinetic models, whereas equilibrium adsorption was fitted using Langmuir, Freundlich, Redlich–Peterson, and Sips isotherm models. Results showed that higher 2,4-D adsorption capacities were found for the chitosan-based hydrogel (64.34 mg g^−1^) compared to the chitosan/magnetite-based composite hydrogel (43.95 mg g^−1^) as a result of physicochemical interactions between iron atoms in magnetite and the hydrogel network.

Functionalized imidazole coordination complexes have been tested as adsorbents by Mansab and Rafique [213] for 2,4-D. It was found that the amino-functionalized complexes (Zn(NH_2_Hiba)_2_) favored Langmuir isotherm while Freundlich isotherm was best fitted for non-functionalized complexes (Zn(Hiba)_2_) and acetic anhydride-functionalized coordination complex (Zn(NH-Hiba)_2_COCH_3_). The Langmuir maximum adsorption capacities calculated for (Zn(Hiba)_2_), (Zn(NH_2_Hiba)_2_), and (Zn(NH-Hiba)_2_COCH_3_) were 12.7, 13.0, and 43.1 mg g^−1^, respectively. Adsorption kinetics followed the PSO model. Thermodynamic studies indicated the spontaneous physisorption process has an endothermic nature. 

Metal-organic framework, a porous chromium-benzenedicarboxylate called MIL-53, was examined as a potential adsorbent for the removal of 2,4-D from an aqueous solution [214]. The kinetics of the 2,4-D adsorption was interpreted with a PSO model, and the adsorption isotherm was described using the Langmuir equation. The maximum monolayer adsorption capacity was reported as 556 mg g^−1^.

A similar material—MIL-101(Cr) metal-organic framework was synthesized and characterized, and its adsorption capacity for MCPA in aqueous solutions was investigated in a paper by Isiyaka et al. [215]. The batch experiments showed that the kinetics, isotherm, and thermodynamics of the adsorption process were described by the PSO, Freundlich, and endothermic adsorption, respectively.

Another metal-organic framework—amino-functionalized zirconium MOF (UiO-66-NH_2_) was prepared by Wei et al. [216] to remove MCPA from aqueous solutions. In that investigation, adsorption kinetics, adsorption isotherms, thermodynamics, and adsorbent regeneration were studied. The effects of adsorbent dosage, pH value, and ionic strength were also investigated. The adsorption of MCPA on UiO-66-NH_2_ was very fast and followed PSO kinetics. The adsorption process was well described by the Langmuir isotherm, and the maximum adsorption capacity was found to be 300.3 mg g^−1^.

Recently, Isaeva et al. [217] investigated the removal of 2,4-D by metal-organic frameworks of the MIL type, such as NH_2_-MIL-53(Al), MIL-53(Al), and NH_2_-MIL-101(Al), and, additionally, for comparison, micro- and mesoporous carbon matrices with different textural characteristics and functional groups. Results revealed that the adsorption efficiency is affected by the texture and functionality of the materials, whereas in the case of MOFs, the adsorption efficiency is also under the influence of framework flexibility. 

The adsorption of 2,4-D onto iron-based metal-organic framework MOF-MIL-100(Fe) has been studied [218]. The adsorption equilibria were fitted by the Langmuir, Freundlich, Sips, Dubinin–Radushkevich, and Redlich–Peterson isotherm models, with a monolayer adsorption capacity for 2,4-D of 858 mg g^−1^. The adsorption kinetics were best described by the PSO equation. The effects of solution pH and ionic strength as well as temperature on the adsorption were investigated. The results revealed that the dominant interaction was an electrostatic attraction between deprotonated 2,4-D and positively charged MIL-100(Fe) and that the process was spontaneous.

In a recent study, Liu et al. [219] prepared Fe-Zr-based metal-organic frameworks (Zr doped MIL-101(Fe)). The material was found to have a featured porous structure and a high surface area of 1003 m^3^ g^−1^. The authors found zirconium doped MIL-101(Fe) can be used for the removal of 2,4-D with maximum adsorption capacity at 357.14 mg g^−1^. Results showed that the adsorption process was consistent with the Langmuir model and pseudo-second-order model.

Zinc ferrite nanoparticles (ZnFe_2_O_4_) were prepared by the sol-gel combustion method and tested for 2,4-D removal [220]. The effects of various experimental conditions, such as solution pH, ZnFe_2_O_4_ dose, initial concentration of 2,4-D, contact time, ionic strength, and temperature, were studied. The PFO and PSO as well as the Langmuir and Freundlich equations were used to analyze the kinetic and equilibrium data, respectively. The results revealed that the PSO and Freundlich models have a better fit than the PFO and Langmuir, respectively.

Mohammadi et al. [221] published a research article that presented data about the adsorption of 2,4-D and MCPA on amine-modified magnetic nanoparticles (Fe_3_O_4_@SiO_2_@NH_2_). Adsorbent efficacy was studied by investigating the effect of contact time, pH, initial concentration of pollutants, adsorbent dose, and temperature. The results from the study of adsorption kinetics showed that the process followed the PSO model. The equilibrium data were fitted to the Langmuir model, giving the maximum adsorption capacity of 166.6 mg g^−1^ for 2,4-D and 153.8 mg g^−1^ for MCPA.

The magnetic hydrochar composite (Fe@PWHC) was produced via hydrothermal carbonization of pomegranate waste and tested for the uptake of 2,4-D from water [222]. The results showed that the Freundlich model explained the adsorption process better compared to the Langmuir model. A maximum adsorption capacity was found to be 101.10 mg g^−1^. Thermodynamic studies revealed the endothermic spontaneous adsorption of 2,4-D.

Valente et al. [223] fabricated β-cyclodextrin-based nanosponges (CD-NSs) synthesized using diamines with 6 and 12 methylene groups (CDHD6 and CDHD12) for adsorptive removal of 2,4-D from polluted water. The maximum adsorption for CDHD6 and CDHD12 was obtained at pH 4.0 and 3.0, respectively. The kinetic data were fitted using the PFO, PSO, Elovich, and Weber-Morris kinetic models, and the best fit was obtained for the Elovich and the pseudo-first-order models. The adsorption isotherm for CDHD12 was well described by the Henry model, while the best model to describe isotherm for CDHD6 was obtained by the additive superposition of the models of Langmuir and Sips.

Very recently, another research team examined the removal of bisphenol A and 2,4-D by lignin immobilized β-cyclodextrin composite microspheres fabricated by a reverse-phase suspension method [224]. Adsorption kinetics, adsorption isotherms, and thermodynamics were investigated. According to the Langmuir model, the adsorption capacity was found to be 497.6 mg g^−1^.

Table 10 presents the compilation of results of various unconventional adsorbents in the removal of chlorophenoxy herbicides.

The materials presented in Table 5, Table 6, Table 7, Table 8, Table 9 and Table 10 are of various origins, prepared in different ways, and differ in their porosity and surface chemistry. Therefore, it is difficult to compare them and their ability to adsorb given herbicides, and the adsorption mechanisms may also be quite different.

## 6. Conclusions

This review discusses the physical, chemical, and biological properties of phenoxycarboxylic acid herbicides and the main sources of their presence in the environment. The main topic of this review is the removal of chlorophenoxy herbicides from the aquatic environment using the adsorption process. It is now accepted that adsorption is one of the best procedures used to remove organic contaminants from water. In the first section of the paper, the various types of conventional and unconventional carbonaceous adsorbents used for this purpose are discussed. The carbon adsorbents discussed are classified as commercial activated carbons, activated carbons from solid wastes, and other carbonaceous materials (carbon blacks, carbon nanotubes, graphene, reduced graphene oxide, and ordered mesoporous carbons). In the further section, inorganic adsorbents and various unconventional adsorbents are presented. The first group consists of inorganic adsorbents that have long been known and used in adsorption processes. These mainly include silica gels, activated alumina, zeolites, molecular sieves, and metal oxides. The second large group consists of various alternative, low-cost adsorbents that include natural materials, biosorbents, and industrial by-products. The suitability of each group of adsorbents for the removal of chlorophenoxy herbicides from the aquatic environment is evaluated. Their effectiveness as adsorbents and their availability and price are taken into account. In the literature on the subject under discussion, the most commonly used phenoxy herbicides are 2,4-D and MCPA. Studies revealing adsorption kinetics and adsorption isotherm fitting are presented. The issues discussed should facilitate the optimal selection of adsorbent type and process conditions.

Summing up the studies presented in this review, some perspectives for future research can be formulated based on the limitations of previous investigations and predicted directions of development. Taking into account the complexity of natural systems, the studies on adsorption technique should be to a larger extent widened to multicomponent systems, including, for example, the adjuvants in herbicide preparations and other organic and inorganic substances of divergent toxicity commonly occurring in waters and soils. Thus, on the one hand, research should be focused on obtaining the selective materials effective for some specific substances, and, on the other hand, on a synthesis of the universal adsorbents showing strong affinity towards various groups of compounds. Moreover, taking into account two main factors determining the adsorption effectiveness, adsorption capacity and rate, experimental and theoretical investigations on adsorption kinetics in multicomponent systems should, in particular, be developed. Another problem is connected with a limited range of studies on the possibility of the reuse of applied adsorbents, the methods of their cyclic cleansing, and the eventual recovery of valuable substances. Moreover, regeneration is very important from an economic and environmental point of view. Recovery of the adsorbate and subsequent regeneration and reuse of the adsorbent reduces the need for new adsorbent and also reduces the problem of the disposal of used adsorbent. In general, there are many regeneration techniques, such as thermal regeneration, steam regeneration, acid or alkaline regeneration, and solvent regeneration. The choice of a particular regeneration method should depend on the physical and chemical properties of both the adsorbate and the adsorbent. Unfortunately, such regeneration is also expensive, if only because it requires energy or the use of suitable, often expensive, reagents. Therefore, when considering the cost of the overall adsorption process, it is necessary to decide whether such regeneration is cost effective. In our work, we have investigated various adsorbents, both conventional and unconventional. Unconventional adsorbents, often commonly referred to as ‘low-cost’ adsorbents, are various types of materials that are not only cheap but also highly available and generally do not require regeneration. A large group of these adsorbents consists of various types of natural materials and industrial, agricultural, and household wastes. Regeneration and recovery of such ‘waste’ is not economical and is rarely reported in the literature. Adsorbent regeneration makes sense when using expensive and highly efficient adsorbents, such as activated carbons. However, although there is no doubt that regeneration is a very important aspect of adsorption, it is not often described in the literature. For this reason, a comparative study on the reuse of the adsorbents that are presented in this review seems to be impossible, and there is simply too little data for a full comparison and analysis. This leads to an important conclusion that studies in this direction should be intensified in the future.

## Figures and Tables

**Table 1:** Physical and chemical properties of phenoxyacetic acid herbicides.

Parameter	4-CPA	2,4-D	2,4,5-T	MCPA	Ref.
Molecular formula/ structure	C_8_H_7_ClO_3_ 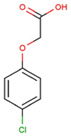	C_8_H_6_Cl_2_O_3_ 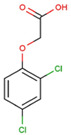	C_8_H_5_Cl_3_O_3_ 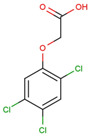	C_9_H_9_ClO_3_ 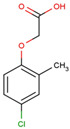	
Molecular weight, g mol^−1^	186.59	221.04	255.48	200.62	
Ionization constant, (pK_a_)	3.14	2.80 2.81	2.56 2.85	3.36 3.07	[23,24,25]
Solubility, g dm^−3^ at 20–25 °C	0.848 0.957	0.682 0.900	0.268 0.278 0.281 0.280	0.825 0.734	[25,26,27,28]
K_ow_ (logP)	1.85 3.2	2.37	2.89 3.13	3.25	[29,30]
D_min_/D_max_, Å	4.33/9.38	4.88/9.49	6.22/8.88	5.47/9.52	[31]
Melting/boiling point, °C	157–158/160	136–140/160	157–158/ above 200 (decomposition)	108–112/ 160 118–119	[26,27,28]
Vapour pressure, Pa at 20–25 °C	9 × 10^−2^	1 × 10^−5^ 2 × 10^−5^	0.7 × 10^−6^	2.3 × 10^−5^ 2 × 10^−4^	[25,27,28]

where: pK_a_—values based on partial charge distribution in a molecule; log D—octanol-water coefficient at a given pH; log P—partition coefficient of a compound between octanol/water; D_min_, D_max_—measure between the most distant molecule atoms.

**Table 2:** The results of chlorophenoxy herbicides adsorption studies on commercial activated carbons.

Activated Carbon	S_BET_, m^2^ g^−1^	Adsorption Capacity (q_m_), mg g^−1^		Isotherm Model	Kinetic Model	Ref.
		2,4-D	MCPA			
F400 (Chemviron, Feluy, Belgium) (water washed)	No data	-	388	L	-	[88]
F400 (Calgon Co, Moon Township, PA, USA) SLS103(Calgon Co) WWL (Calgon Co) (washed with distilled water)	800 1040 670	135.9 142.8 126.2	-	L, F, S	k_f_, D_s_, D_p_, k_s_, D_L_, D_m_	[91]
Norit 0.8 Aquacarb 207C Aquacarb 208A Aquacarb 208EA	1150 ~1100 ~1125 ~1175	-	133.6 117.6 106.2 105.3	L, F, D-R	D_eff_, β_L_	[76]
Nuchar WV-H (Westvaco, Covington, GA, USA) (washed with distilled water)	600–650	555.5	-	F, L, R-P, K-C	PFO, PSO, k_1_,k_2_,W-M, k_L_, K,	[77]
PAC Sigma C-5510 (Aldrich Co, St. Louis, MI, USA) (washed with phosphoric and sulfuric acid)	750	333.3	-	F, L, R-P	PFO, PSO, W-M	[94]
F400 (Calgon Co)	800	-	389.2	L, F, S	k_f_, D_s_, D_p_, Bi, D_L_, D_m_	[78]
F400 (Chemviron) F400AN (washed and HT in H_2_ 3 h 1173 K)	790 960	8.11 11.8	-	L, F	PSO, B, k_2_, D_i_	[92]
F400 (Calgon Co)	800	137.0	108.3	L, F, S	k_f_, D_s_, D_p_, Bi, D_L_, D_m_	[79]
F300 (Calgon Co) (non-modified)	731.5	181.8	-	L, F	PFO, PSO	[81]
F300 (Chemviron) (washed with boiling water)	1098	-	-	L-F	m-exp, PSO	[72]
Sorbo Norit Ceca AC40	1225 1201	329.3 344.8	417.2 521.6	L, F	-	[82]
exp. activated carbon RIB from Norit, washed with conc. HCl, 60 °C, 6 h	1190	-	-	GL	MOE, f-MOE, IDM, PDM	[95]
Norit R3ex (deashed) L2S (Ceca, La Garenne-Colombes, France)	1530 925	340.3 194.5	-	-	PFO, PSO	[83]
AG (Gryfskand, Gryfin, Poland): G0, G33 (after 33%wt abrasion), G66 (after 66%wt abrasion)	800 770 740	-	-	GL, L-F	m-exp, f-MOE, McKay	[73]
GAB CBP (washed with boiling water)	1189 1288	367.1 273.1	599.9 399.9	L, F	-	[93]
F300 (washed 8 h with boiling distilled water)	762	815.6	-	GL, GF, L, T	PFO, PSO, MOE, f-MOE	[23]
Norit SX2 F300	885 965	180.3 191.2	-	F, L	-	[84]
Norit R3ex (deashed) Norit R3ex ox. with conc. HNO_3_ Norit R3ex heated 900 °C (NH_3_)	1390 1296 1212	-	380.3 276.0 542.2	L, F	PFO, PSO	[71]
Norit SX2 F400 Norit R3ex (deashed and HT1800)	870 995 550	269.8 352.7 233.6	-	F, L	PFO, PSO	[96]
Norit R3ex (deashed and HT1800)	554	365.1	-	F, L	PFO, PSO	[97]
Powdered PAC1	630	23.95	-	L, F	-	[87]
Powdered PAC2	568	36.95	-	L, F	-	[87]
CAL 12 × 40 (Calgon Co)	1893	100.1	-	L, F	contact time	[86]

**Table 3:** The results of chlorophenoxy herbicides adsorption studies on activated carbons from solid wastes.

Adsorbent	S_BET_ m^2^ g^−1^	Adsorption Capacity (q_m_), mg g^−1^		Isotherm Model	Kinetic Model	Ref.
		2,4-D	MCPA			
AC from date stones	763.4	238.1	-	L, F, Te	PFO, PSO	[99]
AC from oil palm frond	-	352.9	-	L, F	PFO, PSO	[98]
AC from corncob	1274	300.2	-	L, F	PFO, PSO, W-M, Boyd	[101]
AC from banana stalk	-	196.3	-	L, F	PFO, PSO	[100]
AC from pumpkin seed hull	737.9	260.8	-	L, F, Te	PFO, PSO	[102]
AC from coconut shell	986.2	368.0	-	L, F, Te	PFO, PSO, E	[104]
AC from langsat empty fruit bunch	1070	261.2	-	L, F	PFO, PSO	[103]
Waste slurry AC	710	212	-	L, F	PFO, PSO, Bangham	[105]
AC from a waste PET	1334	-	298.9	L, F	Contact time	[107]
Oxidized AC from PET	885	-	182.6	L, F	Contact time	[107]
Temperature-modified AC from PET	604	-	214.7	L, F	Contact time	[107]
NaOH-modified AC from PET	1110	-	441.4	L, F	Contact time	[107]
Urea modified AC from PET	1167	-	491.5	L, F	Contact time	[107]
AC from particleboard	1211	302.8	375.1	L, F	Contact time	[108]
AC from medium-density fiberboard	1195	263.0	292.9	L, F	Contact time	[108]
AC from PET (K_2_CO_3_)	1206	430.9	491.5	L, F	Contact time	[106]
AC from PAN (KOH)	2828	550.3	527.6	L, F	Contact time	[106]

**Table 4:** The results of chlorophenoxy herbicides adsorption studies on other carbon materials.

Adsorbent	S_BET_ m^2^ g^−1^	Adsorption Capacity (q_m_), mg g^−1^		Isotherm Model	Kinetic Model	Ref.
		2,4-D	MCPA			
SRB N762 carbon black	24.3	6.7	5.8	L	-	[109]
CA N660 carbon black	36.0	21.2	18.9	L	-	[109]
CO N539 carbon black	39.4	24.3	23.0	L	-	[109]
CA N375 carbon black	90.4	52.6	44.9	L	-	[109]
CO N375 carbon black	94.6	54.7	48.2	L	-	[109]
CA N115 carbon black	137.0	41.6	37.1	L	-	[109]
Carbopack B carbon black	97	68.3	-	L, F	PFO, PSO, W-M	[110]
Vulcan XC 72 carbon black	227	71.8	-	L, F	PFO, PSO, W-M	[110]
Carbopack B carbon black	98	34.5	-	-	PFO, PSO	[83]
Vulcan XC 72 carbon black	230	63.2	-	-	PFO, PSO	[83]
Carboxen 1000 carbon molecular sieve	1200	199.4	-	-	PFO, PSO	[83]
Carboxen 1021 carbon molecular sieve	600	94.2	-	-	PFO, PSO	[83]
Carbon black	1085	64.0	-	L, F	-	[111]
N-220 carbon black	108	30.5	36.3	L, F, Te	PFO, PSO, W-M, B	[112]
H_2_O_2_ oxidized carbon black	95	27.4	27.9	L, F, Te	PFO, PSO, W-M, B	[112]
APTES-modified carbon black	82	75.2	69.8	L, F, Te	PFO, PSO, W-M, B	[112]
MWCNT	200	21.87	-	-	-	[114]
SWCNT	700	192.3	-	L, F	-	[116]
SWCNT	597	442.3	-	L, F	-	[97]
rGO	512	270.1	-	L, F	-	[97]
Graphene nano sheets	-	295.8	411.1	L, F, Te, D-R	PFO, PSO, E, W-M	[117]
Aminosilane grafted mesoporous carbons C_KIT-6_	834	110	-	L, F	PFO, PSO	[118]
Carbon materials from filter paper	182.4	77	-	L, F, Te, R-P, To, L-F	PFO, PSO, E	[119]
Carbon materials from cotton	27.4	33	-	L, F, Te, R-P, To, L-F	PFO, PSO, E	[119]
Modified activated carbon fiber	743.3	555.6	-	L, F, Te, D-R	PFO, PSO, W-M	[120]
Fe/OMC	882	300.4	-	L, F, Te	PFO, PSO	[121]
Carbon-SBA-15 replica	739	175.4	-	L, F, Te, D-R	PFO, PSO, W-M	[122]
Magnetic Fe_3_O_4_@graphene nanocomposite	-	32.3	-	L, F	PFO, PSO	[123]
GO-Fe_3_O_4_	-	67.3	-	L, F, Te	PFO, PSO, W-M, B	[124]
3D/GO/Fe_3_O_4_	-	5.26	-	L, F, Te, R-P	PFO, PSO, W-M	[125]
Activated charcoal/Fe_2_O_3_ nanocomposite	560.1	255.1	-	L, F, Te, D-R	PFO, PSO, E, W-M	[126]
Graphene oxide/MIL 101(Cr)	-	476.9	-	L, F	PFO, PSO	[127]
Activated carbon fiber—Fe_3_O_4_	-	51.10	-	L, F, Te	PFO, PSO, W-M	[128]
Adsorbent from elutrilithe A(700)	122.4	-	32.1	L	PSO	[129]
Adsorbent from elutrilithe A(800)	119.4	-	30.7	L	PSO	[129]
Adsorbent from elutrilithe A(900)	161.9	-	38.7	L	PSO	[129]

**Table 5:** The results of chlorophenoxy herbicides adsorption studies on inorganic materials.

Adsorbent	BET m^2^ g^−1^	Adsorption Capacity (q_m_) mg g^−1^		Isotherm Model	Kinetic Model	Ref.
		2,4-D	MCPA			
MCM-41	572.7	2.48	-	L	-	[137]
APTES modified MCM-41	173.8	187.3	-	L	-	[137]
SBA-15	722	40.7	-	L, F	PSO	[133]
APTES modified SBA-15	511	278	-	L, F	PSO	[133]
TMSPU modified SBA-15	498	70.9	-	L, F	PSO	[133]
MCF	593	31.6	-	L, F	PSO	[133]
APTES modified MCF	415	286	-	L, F	PSO	[133]
TMSPU modified MCF	410	57.1	-	L, F	PSO	[133]
APTES-modified silica gel	-	10.32	-	L	-	[139]
Molecularly-imprinted APTES-modified silica gel	185	125	-	-	Contact time	[140]
APTES-modified silica gel	355	50	-	-	Contact time	[140]
Aluminum-based metal-organic framework	1104	-	231.9	F	PFO, PSO, W-M	[141]

**Table 10:** The results of chlorophenoxy herbicide adsorption studies on various unconventional materials.

Adsorbent	BET m^2^ g^−1^	Adsorption Capacity (q_m_) mg g^−1^		Isotherm Model	Kinetic Model	Ref.
		2,4-D	MCPA			
Magnetite-GO-LDH composites	74.9	173	-	L, F	PFO, PSO	[209]
Red mud@carbon composite	89.5	111.1	-	L, F, R-P	PFO, PSO, W-M	[210]
Fe-crosslinked chitosan complex	-	473	-	L	PFO, PSO,	[211]
Chitosan-based hydrogel	-	64.34	-	L, F, R-P, S	PFO, PSO, E	[212]
Chitosan/magnetite-based composite hydrogel	-	43.95	-	L, F, R-P, S	PFO, PSO, E	[212]
Zn(Hiba)_2_ complexes	409.1	12.7	-	L, F	PFO, PSO, W-M	[213]
Zn(NH_2_Hiba)_2_ complexes	256.7	13.0	-	L, F	PFO, PSO, W-M	[213]
Zn(NH-Hiba)_2_COCH_3_ complexes	183.1	43.1	-	L, F	PFO, PSO, W-M	[213]
MOF-MIL-53	1438	556	-	L	PSO	[214]
MOF-MIL-101(Cr)	1439	-	370.4	L, F, Te	PFO, PSO, W-M	[215]
MOF-UiO-66-NH_2_	694	-	300.3	L, F	PFO, PSO	[216]
MOF-NH2-MIL-53(Al)	79	241	-	-	contact time	[217]
MOF-MIL-53(Al)	633	336	-	-	contact time	[217]
MOF-NH_2_-MIL-101(Al)	2895	172	-	-	contact time	[217]
MOF-MIL-100(Fe)	1893	858	-	L, F, S, D-R, R-P	PFO, PSO, W-M, Ba	[218]
Zr-MOF-MIL-101(Fe)	1003	357.1	-	L, F	PFO, PSO	[219]
ZnFe_2_O_4_ nanoparticles	-	48.30	-	L, F	PFO, PSO	[220]
Fe_3_O_4_@SiO_2_@NH_2_	-	166.6	153.8	L, F	PFO, PSO	[221]
Fe@PWHC	1893	100.1	-	L, F	-	[222]

## Data Availability

The data are available by the corresponding author.

## Abbreviations

AC	Activated carbon
B	Boyd kinetic model
Ba	Bangham kinetic model
Bi	Biot number
β_L_	External mass transfer coefficient, cm s^−1^
D-R	Dubinin-Radushkevich isotherm
D_eff_	Effective diffusivity, cm^2^ s^−1^
D_i_	Effective diffusion coefficient, m^2^ s^−1^
D_L_	Axial dispersion coefficient, m^2^ s^−1^
D_m_	Molecular diffusion coefficient, m^2^ s^−1^
D_p_	Effective pore diffusion coefficient, m^2^ s^−1^
D_s_	Effective surface diffusion coefficient, m^2^ s^−1^
E	Elovich isotherm
F	Freundlich isotherm
f-MOE	Fractal mixed-order equation
F-S	Fritz-Schlunder isotherm model
GF	The Generalized Freundlich isotherm
GL	The Generalized Langmuir isotherm
H	The linear Henry’s Law isotherm
IDM	Intraparticle diffusion model
K-C	Koble-Corrigan equilibrium model
k	Intraparticle diffusion rate in W-M kinetic model, $\frac{mg}{g}\sqrt{\frac{1}{min}}$
k_1_	Rate constant for PFO kinetic model, min^−1^
k_2_	Rate constant for PSO kinetic model, g mg^−1^ min^−1^
k_f_	Film mass transfer coefficient, m s^−1^
k*_s_*	Solid mass transfer coefficient, s^−1^
k_L_	External mass transfer coefficient, cm min^−1^
L	Langmuir isotherm
L-F	Langmuir-Freundlich isotherm
McKay	McKay’s pore diffusion model
m-exp	Multi-exponential kinetic equation
MOE	Mixed-order equation
M-P	McKay and Paterson’s equations
PDM	Pore diffusion model
PFO	Pseudo-first-order kinetic model/equation (PFOE)
PSO	Pseudo-second-order kinetic model/equation (PSOE)
q_m_	The maximum adsorption capacity, mg g^−1^
R-P	Redlich-Peterson isotherm
S	Sips isotherm
Te	Temkin isotherm
To	Toth isotherm
W-M	Weber-Morris kinetic model
